# An artificial nickel chlorinase based on the biotin–streptavidin technology†

**DOI:** 10.1039/d3cc05847f

**Published:** 2024-01-22

**Authors:** Kun Yu, Kailin Zhang, Roman P. Jakob, Timm Maier, Thomas R. Ward

**Affiliations:** a Department of Chemistry, University of Basel Mattenstrasse 22 Basel CH-4058 Switzerland thomas.ward@unibas.ch; b Biozentrum, University of Basel Spitalstrasse 41 Basel CH-4056 Switzerland

## Abstract

Herein, we report on an artificial nickel chlorinase (ANCase) resulting from anchoring a biotinylated nickel-based cofactor within streptavidin (Sav). The resulting ANCase was efficient for the chlorination of diverse C(sp^3^)–H bonds. Guided by the X-ray analysis of the ANCase, the activity of the artificial chlorinase could be significantly improved. This approach opens interesting perspectives for late-stage functionalization of organic intermediates as it complements biocatalytic chlorination strategies.

## Abstract 2

Herein, we report on an artificial nickel chlorinase (ANCase) resulting from anchoring a biotinylated nickel-based cofactor within streptavidin (Sav).

## Graphical abstract

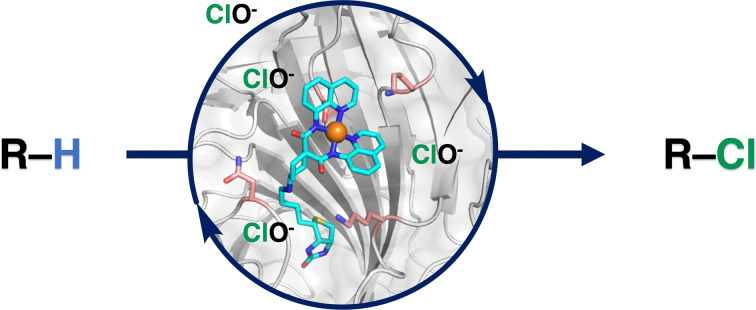

Halogen-containing motifs are ubiquitously present in natural products due to the bioavailability of halides in the environment.^1^ The incorporation of a halogen atom can significantly modulate the properties of a molecule, potentially influencing its bioactivity, metabolism, and pharmacokinetic profile.^2^ Accordingly, halogenated compounds play an important role in both the pharmaceuticals^3^ and agrochemicals sectors,^4^Scheme 1a. Moreover, halogenated compounds can serve as versatile substrates in a myriad of coupling reactions, thus facilitating the synthesis of valuable advanced synthetic intermediates.^5^ Noteworthily, chlorinated natural products dominate the halogenated compound landscape with over 1000 examples.^6^ Therefore, developing efficient chlorination methodologies has attracted increasing attention in recent decades.

**Scheme 1:**
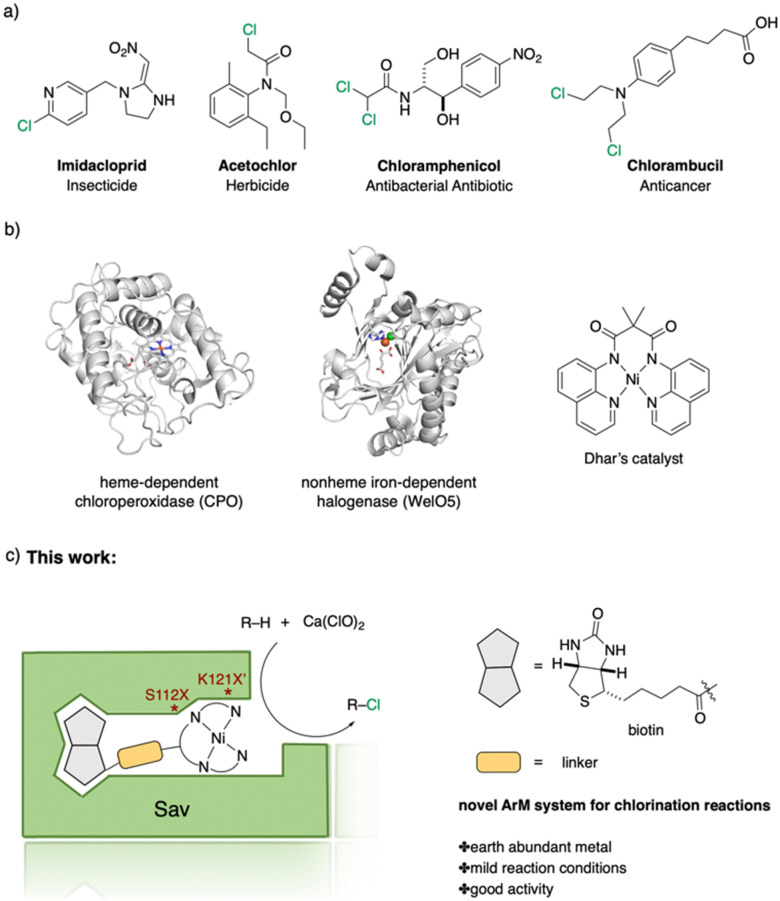
Selected examples of chlorine-containing compounds and recently-reported Ni-catalyst for chlorination reactions. (a) Selected examples of chlorine-containing agrochemicals and pharmaceuticals. (b) Selected examples of enzymes and homogeneous catalyst for chlorination. (c) Artificial Nickel Chlorinase (ANCase) catalyzed chlorination of alkanes.

Given the ubiquity of chlorinated natural products in the environment, the initial research endeavors focused on elucidating the biosynthetic pathway underpinning these molecules. To date, four distinct classes of halogenases have been identified:^7^ heme-/vanadium-dependent haloperoxidases, flavin-dependent halogenases, nonheme iron-dependent halogenases and *S*-adenosyl-l-methionine (SAM)-dependent halogenases. Among them, the α-KG-dependent nonheme iron halogenase, heme-dependent haloperoxidases, and non-heme halogenases SyrB2 offer versatile means to chlorinate inert C(sp^3^)–H bonds, Scheme 1b.

Recently, several groups reported their achievements in utilizing repurposed halogenases.^8^ Benefiting from the development of directed evolution technologies, the substrate scope of chlorination has been significantly expanded. Drawing inspiration from natural chlorinases, chemists have developed several metal complexes that catalyze chlorination reactions. To cite a few, Mn-,^9^ Fe-^10^ and Ni-based^11^ complexes have been reported to be efficient catalysts. Importantly, however, competition between the formation of chlorinated products and oxygenated side-products persists. Recently, the Dhar group reported on Ni-based complexes relying on tetradentate amido-quinoline as a ligand, which exhibited promising chlorination activity, Scheme 1b.

Artificial metalloenzymes (ArMs) result from incorporating a metallocofactor within a host protein. Over the years, ArMs have been shown to combine the advantages of both homogeneous and enzymatic catalysts and endow the protein with new-to-nature reactivity. Since the foundational study by Wilson and Whitesides in the late 1970s,^12^ many research groups have contributed to the development of ArMs displaying diverse catalytic properties.^13^ Building upon the biotin-Sav technology, our team and others^14^ have developed various ArMs. The pronounced affinity of biotin for Sav ensures the tight binding of a biotinylated metallocofactor to the Sav scaffold. Depending on the biotinylated metallocofactor, the assembled ArMs exhibit a diverse range of activities. Herein, we report on an artificial nickel chlorinase (ANCase) for the C(sp^3^)–H chlorination of alkanes, taking advantage of the biotin-Sav technology, Scheme 1c.

Inspired by the chlorination homogeneous catalyst recently reported by Dhar and coworkers, we set out to synthesize a related biotinylated Ni-complex.

To maintain the *C*_2_-symmetry of the metallocofactor, we set out to attach the biotin anchor to the bridging carbon atom flanked by the two amido-quinoline moieties. Additionally, we converted the original dimethyl group to a cyclohexyl entity to minimize the influence on the dihedral angle between the two amido-quinoline planes. Accordingly, the cofactor [(Biot^C4^-bAQ)Ni] 1 was synthesized, see, Scheme S1 (ESI†).

With the cofactor 1 at hand, we set out to confirm its incorporation within Sav. For this purpose, we assessed the quantitative anchoring of the [(Biot^C4^-bAQ)Ni] 1 into Sav WT by a HABA displacement titration using CD spectroscopy as readout.^15^ The determined dissociation constant was *K*_d_ = 0.29 ± 0.05 μM (see ESI†). Next, we evaluated its activity for the chlorination of cyclohexane. In the absence of Sav, [(Biot^C4^-bAQ)Ni] 1 yielded 21 turnovers (TON), Table 1, entry 1.

**Table 1:** Optimization of reaction conditions for ANCase-catalyzed chlorination of cyclohexane^a^

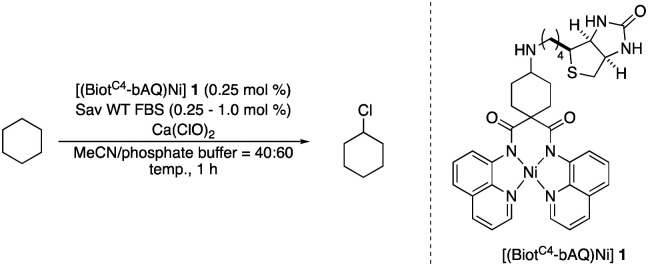					
Entry	[Ni]/Sav FBS	Temperature (°C)	pH	Ca(ClO)_2_ (equiv.)	TON
1	Without Sav	25	9	10	21 ± 2
2	1 : 2	25	9	10	54 ± 11
3	1 : 1	25	9	10	52 ± 8
4	1 : 4	25	9	10	43 ± 7
5	1 : 2	15	9	10	38 ± 1
6	1 : 2	35	9	10	31 ± 7
7	1 : 2	25	6	10	15 ± 3
8	1 : 2	25	8	10	39 ± 5
9	1 : 2	25	10	10	34 ± 2
10	1 : 2	25	9	5	32 ± 4
11	1 : 2	25	9	20	40 ± 7

a Reaction conditions: [cyclohexane] = 20 mM, [(Biot^C4^-bAQ)Ni] 1 = 50 μM, MeCN/phosphate buffer (100 mM) = 200/300 μL, 1 h. Sav WT FBS = streptavidin wild-type free binding sites. Varying amounts of dichlorination products were detected in all cases.

Upon incorporation into Sav WT, [(Biot^C4^-bAQ)Ni] 1·Sav WT hereafter, the resulting ArM afforded significantly higher TON (TON = 54), underscoring the positive influence of the second coordination sphere provided by Sav on chlorinase activity. We then optimized the reaction conditions. Sav is a homotetrameric protein with four free biotin-binding sites (four FBS) consisting of two pairs of close-lying bis-biotin binding sites. In select cases, the occupancy of the adjacent monomers has been shown to affect the catalytic performance of the ArM.^16^

Based on experimental evaluations, we determined the optimal ratio to be [(Biot^C4^-bAQ)Ni] 1/Sav FBS = 1 : 2, Table 1, entries 2–4. Additionally, both temperature and pH substantially influenced TON, Table 1, entries 5–9. The highest TON was obtained at 25 °C and pH = 9. Importantly, an alkaline reaction environment proved essential to minimize the demetallation of the cofactor. The concentration of the hypochlorite markedly influenced TON, Table 1, entry 10 and 11. Striking the right balance in Ca(ClO)_2_ concentration was instrumental in ensuring reaction efficiency. Importantly, across most experiments, only trace amounts of oxygenated side-products were detected, along with dichlorination products. Following the chlorination reaction, we observed the precipitation of Sav. Given the structural similarity of the Ni-cofactor to the iron-porphyrin complex, and considering existing reports on the chlorination of heme-dependent chlorinases,^17^ we hypothesize that Sav may indeed undergo chlorination during the reaction. We attempted to verify this hypothesis by measuring HRMS of the Sav precipitate. Unfortunately, our efforts were impeded by the insolubility of the precipitate: no signal in the corresponding HRMS spectrum could be detected.

Next, we determined the X-ray structure of [(Biot^C4^-bAQ)Ni] 1·Sav WT, Fig. 1 (PDB: 8QQ3). Based on the collected datasets, it is apparent that the [(Biot^C4^-bAQ)Ni] 1 lies close to residues N49, S112, K121, L124 as well as K121′ from the adjacent monomer. Residue N49 establishes hydrogen bonding interaction with the cyclohexyl amine moiety of the cofactor [(Biot^C4^-bAQ)Ni] 1. Residues S112 and L124 collectively constitute a hydrophobic pocket, accommodating the cofactor, see Fig. S2 (ESI†).

**Fig. 1:**
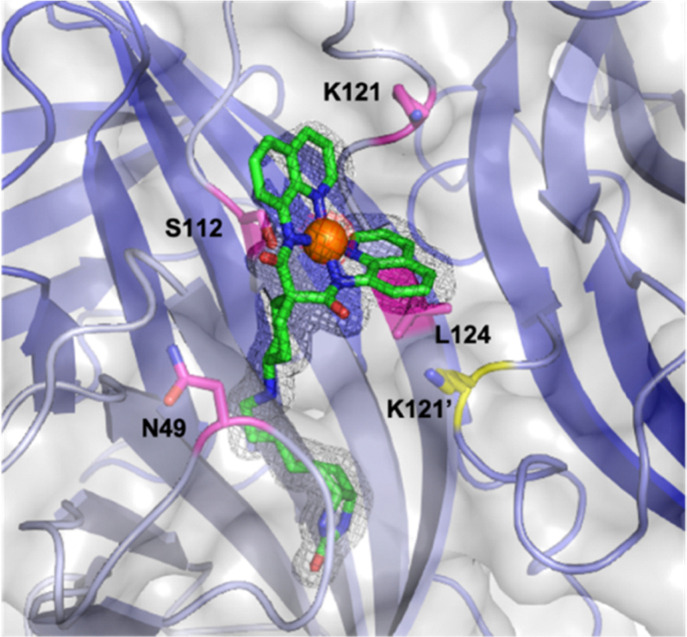
Close-up view of the structure of the [(Biot^C4^-bAQ)Ni] 1·Sav WT (PDB: 8QQ3). The cofactor [(Biot^C4^-bAQ)Ni] 1 is displayed as sticks (atoms are color-coded; nitrogen = blue, oxygen = red, carbon = green or cyan, chloride = green and sulfur = yellow) with the nickel as an orange sphere. The protein is represented as a cartoon and transparent surface model. The monomers are color-coded in different shades of blue. The residues S112 and K121 are displayed as magenta sticks (atoms are color-coded; nitrogen = blue, oxygen = red and carbon = magenta and yellow for the adjacent monomer). The 2F_o_–F_c_ difference map is displayed as a dark grey mesh (1σ) and the anomalous electron density is displayed as red mesh (5σ). The occupancy of the nickel was set to 69%.

Based on the structural information, we performed genetic optimization of [(Biot^C4^-bAQ)Ni] 1·Sav WT targeting positions Sav S112 and K121. Our initial efforts centered on screening single saturation mutagenesis libraries at these two positions, affording thirty-eight corresponding single mutant ANCases. The screening results are presented as a radar plot, Fig. 2a. From this screening, several trends emerged: (i) residues at position K121 exerted a pronounced influence on the activity. (ii) Introducing aromatic residues at position K121, such as Sav K121H, Sav K121F, Sav K121Y, and Sav K121W, leads to enhanced activity. We posit that these substitutions may engage in π–π interactions with the quinoline moiety of the cofactor. Interestingly, Sav K121P also afforded good TON, which could be due to the change in the structure of the protein enforced by the proline. (iii) The effect of mutations at position S112 was less pronounced: slightly higher TONs were obtained with mutants Sav S112D, Sav S112I, and Sav S112R.

**Fig. 2:**
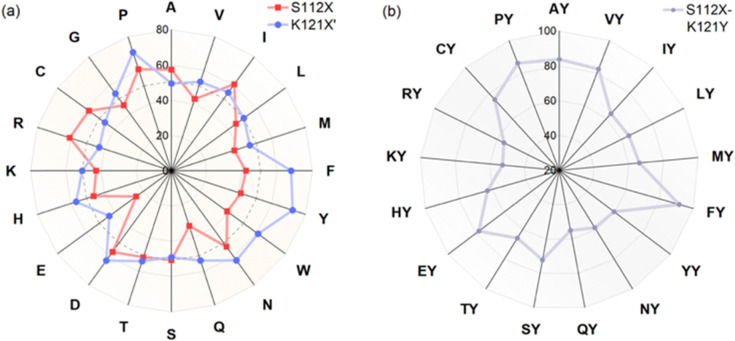
Results for genetic optimization for the chlorination of cyclohexane: TON for reactions using (a) single mutants Sav S112X or Sav K121X′ and (b) double mutants Sav S112X–K121Y are highlighted in red, blue and grey respectively. Double mutants Sav S112X–K121Y are abbreviated as XY in (b).

Considering that the single mutant Sav K121Y gave the highest TON, we fixed this position and saturated position S112. Only sixteen double mutants were obtained after production and affinity purification. These double mutants were evaluated for the chlorination of cyclohexane, Fig. 2b. Several double mutants outperformed their parent ANCase [(Biot^C4^-bAQ)Ni] 1·Sav K121Y. Notably, Sav S112F–K121Y emerged as the most active, affording TON = 92. The double mutant ANCase [(Biot^C4^-bAQ)Ni] 1·Sav S112P–K121Y: TON = 87.

We then attempted to determine Michaelis parameters for [(Biot^C4^-bAQ)Ni] 1·Sav WT and [(Biot^C4^-bAQ)Ni] 1·Sav S112Y. However, all attempts did not reveal a saturation kinetic profile. This suggests that the radical-chlorination mechanism does not proceed *via* the formation of a Michaelis–Menten complex, followed by the rate-determining step. This is consistent with the mechanism suggested by Dhar and coworkers for the related homogeneous catalyst.^11*e*^

Finally, the substrate scope was explored using a focused library of Sav mutants combined with cofactor [(Biot^C4^-bAQ)Ni] 1, Table 2. The ANCases were efficient for the chlorination of diverse C(sp^3^)–H bonds. Key findings from this screening included: (i) for toluene, apart from the expected chlorination products, additional derivatives arising from chlorination of aromatic C(sp^2^)–H bonds were identified. (ii) in the case of ethylbenzene, the dominant product was acetophenone, and a relatively low yield of chlorinated product, *i.e.* 2-(chloroethyl)benzene, was observed. No difference in TON and product distribution was observed for the chlorination of ethylbenzene using [(Biot^C4^-bAQ)Ni] 1·Sav S112F–K121Y upon carrying out the reaction under anaerobic conditions. We hypothesize that the reason for the high yield of oxygenated product is the low oxidation potential of the secondary benzyl radical.^18^ Additionally, no enantioselectivity was achieved in this reaction.

**Table 2:** Substrate scope for the chlorination catalyzed by [(Biot^C4^-bAQ)Ni] 1·Sav variants^a^

Substrate	BDE of the C–H (kcal mol^−1^)^b^	Sav variant	Product	TON^c^
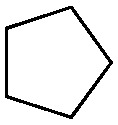	94.9	S112P–K121Y	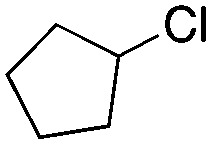	56 ± 2(42)^d^

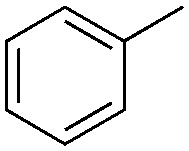	89.6		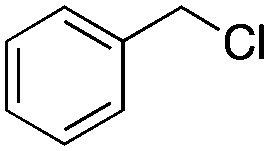	100 ± 7(78)^d^
	110.9	S112F–K121Y	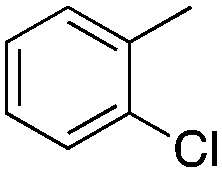	16 ± 4(3)^d^
	111.2		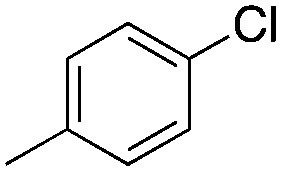	9 ± 2(2)^d^

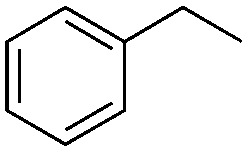	86.9	S112F–K121Y	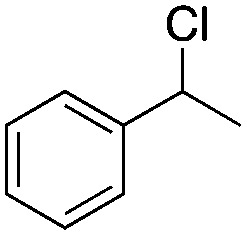	55 ± 4(55)^d^
			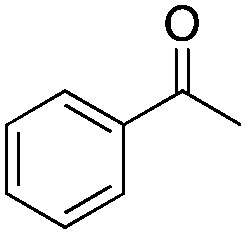	200 ± 2(190)^d^

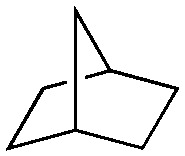	97.2	S112P–K121Y	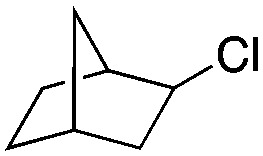	51 ± 3(44)^d^

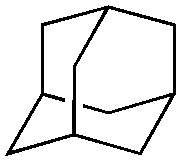	98.8	WT	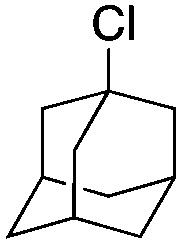	11 ± 1

a Reaction conditions: [substrate] = 20 mM, [Ca(ClO)_2_] = 200 mM, [[(Biot^C4^-bAQ)Ni] 1] = 50 μM, [Sav FBS] = 100 μM, MeCN/phosphate buffer (pH 9, 100 mM) = 200/300 μL, 25 °C, 1 h. Sav FBS = streptavidin free binding sites.

b Bond dissociation energy (BDE) computed with the BDE estimator at https://bde.ml.nrel.gov.

c TONs were determined by GC-MS.

d TON in brackets are from reactions using Sav WT.

In conclusion, we have engineered an ANCase by incorporating the [(Biot^C4^-bAQ)Ni] 1 within a Sav scaffold, thereby catalyzing the chlorination of C(sp^3^)–H bonds. Genetic optimization led to the identification of a double mutant ANCase [(Biot^C4^-bAQ)Ni] 1·Sav S112F–K121Y which was most active for the chlorination of various C(sp^3^)–H bonds. Current efforts focus on achieving regioselective chlorination, which would provide valuable building blocks for organic synthesis.

We thank Lukas Shulte for exploratory studies on ligand synthesis. T. R. W. thanks the support of NCCR catalysis (180544), the NCCR Molecular Systems Engineering (182046) as well as the SNSF (grant 200020_212088). We would like to thank the staff of the Swiss Light Source at the Paul Scherrer Institute for beam time, assistance with crystal testing, and data collection.

## Conflicts of interest

There are no conflicts of interest to declare.

## Supplementary Material

CC-060-D3CC05847F-s001
