# Supplementary material for: An artificial nickel chlorinase based on the biotin–streptavidin technology
Source: Chem Commun (Camb). 2024 Jan 22;60(14):1944–7. doi: 10.1039/d3cc05847f (PMC10863421; doi:10.1039/d3cc05847f)
Supplement: CC-060-D3CC05847F-s001 [file CC-060-D3CC05847F-s001.pdf]

Electronic Supplementary Material (ESI) for ChemComm.
This journal is © The Royal Society of Chemistry 2024

Supporting Information

An Artificial Nickel Chlorinase Based on Biotin-Streptavidin Technology

Kun Yu, [a] Kailin Zhang, [a] Roman P. Jakob, [b] Timm Maier [b] and Thomas R. Ward [a]*

[a] Department of Chemistry, University of Basel, BPR 1096, Mattenstrasse 22, 4058 Basel, Switzerland

[b] Biozentrum, University of Basel, Spitalstrasse 41, 4056 Basel, Switzerland.

Email: thomas.ward@unibas.ch

Table of contents

1. **General aspects** **2**
   1.1. Materials and methods 2
   1.2. Instrumentation 2
2. **Synthesis of cofactors** **3**
   Synthesis of the dicarboxylic acid S2:[1] 3
   Synthesis of the bis-amidoquinoline S3:[2] 3
   Synthesis of the ligand Biot^{C4}-bAQ S4: 4
   Synthesis of the cofactor [(Biot^{C4}-bAQ)Ni] 1:[3] 5
3. **HABA titration for determining the binding constant of [(Biot^{C4}-bAQ)Ni] 1 for Sav WT** **6**
4. **Crystallographic characterization of [(Biot^{C4}-bAQ)Ni] 1 · Sav WT** **8**
5. **Expression and purification of Sav mutants** **10**
   5.1. General procedure for the expression and purification of Sav variants for catalysis 10
   5.2. Sequence of selected Sav muntants 11
6. **Catalysis with purified Sav variants** **12**
   6.1. General procedure for catalysis with purified Sav variants 12
7. **Supporting tables** **15**
8. **Supporting Figures** **20**
9. **NMR Spectra** **35**

1. General aspects

1.1. Materials and methods

All commercially-available chemicals were purchased from Sigma-Aldrich, Acros Organics, TCI Europe, Fluorochem and used without further purification. All substrates and reference products were purchased from Sigma-Aldrich. Dry solvents were purchased from Acros Organics and used directly without further purification. The water used for all biological and catalytic experiments was purified with a Milli-Q Advantage system.

1.2. Instrumentation

^{1}H and ^{13}C spectra were recorded on a Bruker 500 MHz at room temperature. Chemical shifts are reported in ppm (parts per million) relative to TMS (δ = 0.00 ppm for ^{1}H and ^{13}C). Signals are quoted as s (singlet), d (doublet), t (triplet), bs (broad singlet) and m (multiplet). Spectra were analyzed on MestReNova and calibrated relative to the residual solvent peak. Electron-Spray Ionization Mass Spectra (ESI-MS) were recorded on a Bruker FTMS 4.7T bioAPEX II. High-resolution mass spectra (HRMS) were measured on a Bruker maXis 4G QTOF ESI mass spectrometer. GC-MS analysis of catalysis was performed on a Shimadzu GC-2010 Plus system coupled with a GC-MS-QP2020 detector (column: Agilent HP-5 (30 m × 0.25 mm × 0.25 μm)) using He as carrier gas and naphthalene as internal standard. GC analysis of catalysis was performed on a GC-FID equipped with a Astec® CHIRALDEX™ G-TA Capillary GC Column (50 m 0.25 mm 0.12 μm) using He as carrier gas and naphthalene as internal standard. Circular Dichroism (CD) spectra were recorded on a Chirascan from Applied Photophysics at 25 ^{o}C using a quartz cell (1 cm path length). Flash chromatography was performed on a Biotage Isolera or a Buchi Pure chromatography system. Preparative HPLC purifications were carried out with a Water Prep LC 4000 System with an Agilent XDB-C18 column (21.2 × 150 mm, 5 μm). No unexpected or unusually high safety hazards were encountered.

2. Synthesis of cofactors

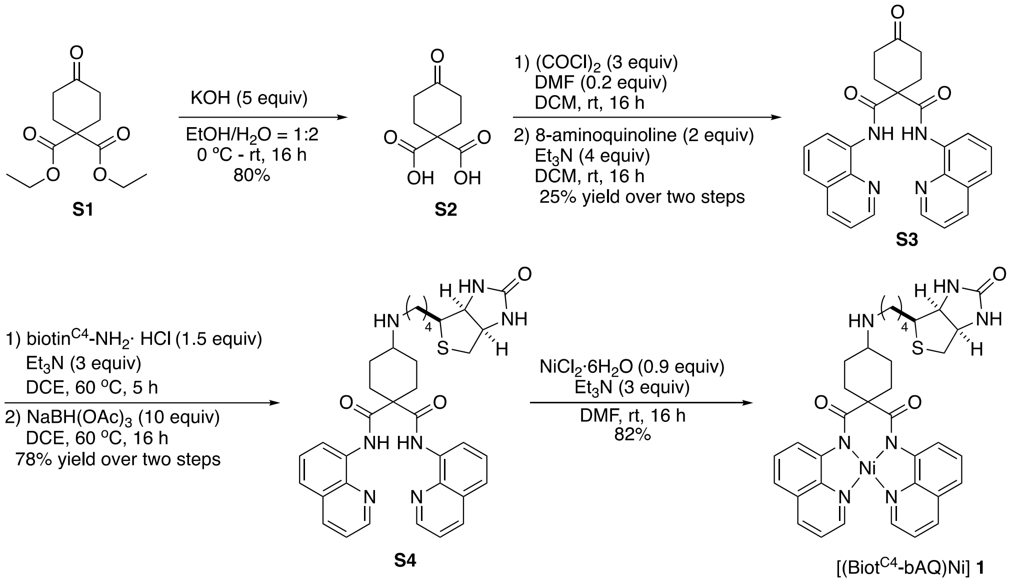

Scheme S1. Synthetic route of the Ni-cofactor [(Biot^{C4}-bAQ)Ni] **1**.

Synthesis of the dicarboxylic acid S2:[1]

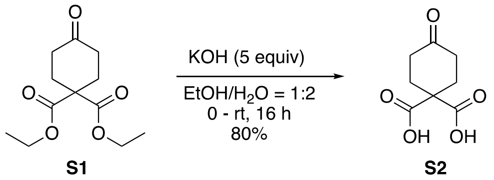

KOH (1.74 g, 31.0 mmol) was added to a solution of the dicarboxylate **S1** (1.50 g, 6.20 mmol) in water/EtOH (4 mL/8 mL). The solution was stirred at 100 °C under reflux for 4 h. The solvent was removed *in vacuo*, and the residue was redissolved in water (20 mL). Then, the solution was acidified using HCl (2 M). The mixture was extracted with diethyl ether (3 × 15 mL). The combined organic layers were washed with brine and dried over anhydrous $MgSO_4$. The solvent was removed *in vacuo* to afford the dicarboxylic acid **S2** (924 mg, 80%) as a white solid.

^{1}H NMR (500 MHz, DMSO-d_6): 2.31 (t, J = 6.8 Hz, 4H), 2.20 (t, J = 6.8 Hz, 4H).

^{13}C NMR (126 MHz, DMSO-d_6): 209.8, 172.7, 53.2, 37.8, 30.7.

HRMS (ESI positive mode, m/z): calculated for $C_8H_{10}O_5$ $[M–H]^-$ 185.0455; found 185.0459.

Synthesis of the bis-amidoquinoline S3:[2]

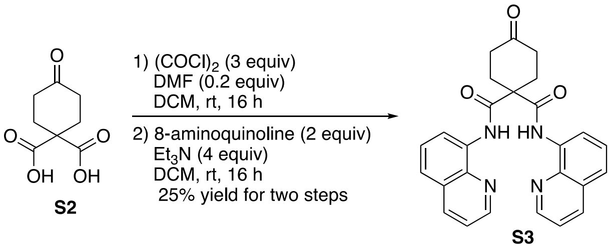

Step 1: Oxalyl chloride (1.22 g, 9.67 mmol) was added to a solution of dicarboxylic acid **S2** (600 mg, 3.22 mmol) and DMF (47 mg, 0.65 mmol) in DCM (10 mL) dropwise at 0 ºC. The reaction mixture was stirred at room temperature overnight. The solvent was removed *in vacuo*. The residue was used for next step directly. **Step 2:** The residue from step 1 was dissolved in dry DCM (10 mL) and then was added dropwise at 0 ºC within 1 h to a solution of 8-aminoquinoline (929 mg, 6.45 mmol) and triethylamine (1.30 g, 19.3 mmol). The reaction mixture was stirred at room temperature overnight. The solution was filtered through a celite pad and concentrated *in vacuo*. The residue was purified by silica gel flash chromatography (DCM/EA = 98/2 – 80/20) to afford the crude bis-amidoquinoline **S3**. A more thorough purification was carried out after the biotinylation step.

Synthesis of the ligand Biot^{C4}-bAQ S4:

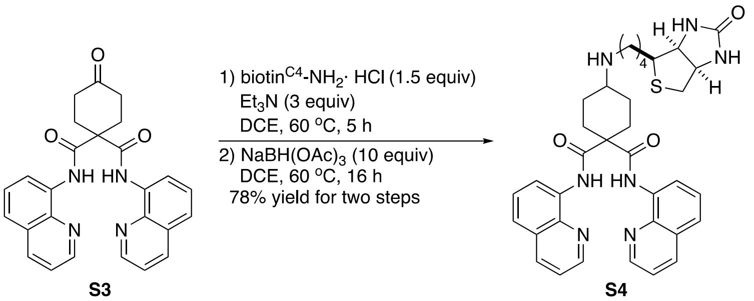

Step 1: Biotin^{C4}-NH_2·HCl[3] (1.5 equiv) was added to a stirring solution of the bis-amidoquinoline **S3** (90 mg, 0.21 mmol, 1.0 equiv) in dry DCE (5 mL). Triethylamine (62.4 mg, 0.62 mmol) was then added. The reaction mixture was stirred at 60 ºC for 5 h. **Step 2:** $NaBH(OAc)_3$ (435 mg, 2.1 mmol) was added to the reaction, and the mixture was stirred at 60 ºC overnight. The white precipitate was removed by filtration, and the filtrate was used for purification by preparative HPLC (Solvents were composed as follows: (A) water/acetonitrile/TFA = 97:3:0.1; (B) acetonitrile/TFA = 99.9:0.1. Method: 0 min – 0% B; 4 min – 10% B; 30 min – 90% B; 31 min – 100% B; 36 min – 100%.).

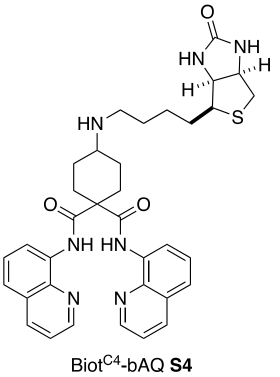

Biot^{C4}-bAQ **S4**

White solid. Biot^{C4}-bAQ **S4** was synthesized following the same procedure in 78% yield (over 2 steps).

^{1}H NMR (500 MHz, DMSO-d_6): 10.64 (s, 1H), 10.55 (s, 1H), 8.95 (dd, J = 4.3, 1.6 Hz, 1H), 8.91 (dd, J = 4.3, 1.7 Hz, 1H), 8.68 (dd, J = 7.7, 1.3 Hz, 1H), 8.61 (dd, J = 7.7, 1.3 Hz, 1H), 8.45 (ddd, J = 12.5, 8.4, 1.7 Hz, 2H), 8.35 (brs, 2H), 7.75 (dd, J = 8.4, 1.3 Hz, 1H), 7.70 (dd, J = 8.4, 1.3 Hz, 1H), 7.69 – 7.65 (m, 2H), 7.65 – 7.62 (m, 1H), 7.59 (t, J = 8.0 Hz, 1H), 4.32 (ddd, J = 7.8, 5.1, 1.0 Hz, 1H), 4.14 (dd, J = 7.8, 4.4 Hz, 1H), 3.97 (s, 1H), 3.24 – 3.22 (m, 1H), 3.11 – 3.08 (m, 1H), 2.97 – 2.90 (m, 2H), 2.83 – 2.81 (m, 3H), 2.59 (d, J = 12.4 Hz, 1H), 2.25 – 2.23 (m, 2H), 2.15 (td, J = 13.8, 3.4 Hz, 2H), 1.72 – 1.53 (m, 5H), 1.48 (m, 1H), 1.37 (m, 2H).

^{13}C NMR (126 MHz, DMSO-d_6): 169.4, 168.8, 163.3, 159.3, 159.0, 158.7, 158.4, 150.0, 149.8, 138.5, 138.3, 137.3, 137.3, 133.8, 133.7, 128.2, 128.2, 127.5, 127.4, 123.4, 123.0, 123.0, 122.9, 117.3, 117.1, 116.6, 115.0, 61. 5, 59.7, 56.6, 55.7, 55.5, 54.9, 49.1, 44.4, 29.8, 28.3, 26.3, 26.0, 25.8, 25.8.

HRMS (ESI positive mode, m/z): calculated for $C_{35}H_{39}N_7O_3S$ $[M+H]^+$ 638.2908; found 638.2895.

Synthesis of the cofactor [(Biot^{C4}-bAQ)Ni] 1:[4]

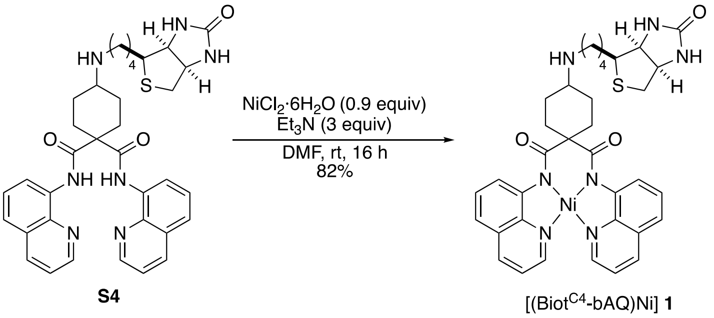

Triethylamine (19 mg, 0.19 mmol, 4.0 equiv) was added to a solution of ligand **S4** (0.047 mmol, 1.0 equiv) in dry DMF (3 mL) under a flow of nitrogen. The mixture was stirred for 15 min at room temperature, and then $NiCl_2·6H_2O$ (10 mg, 0.043 mmol, 1.1 equiv) was added. The mixture was stirred at room temperature overnight. The solvent was removed *in vacuo*. The resulting residue was purified by washing with DCM to afford the corresponding Ni-cofactor [(Biot^{C4}-bAQ)Ni] **1**.

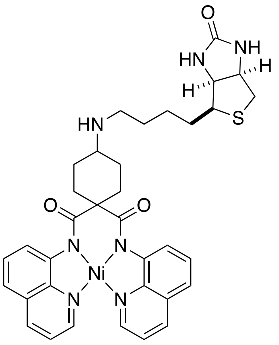

[(Biot^{C4}-bAQ)Ni] **1**

Green solid. [(Biot^{C4}-bAQ)Ni] **1** was synthesized following the general procedure in 82% yield.
^{1}H NMR (500 MHz, DMSO-d_6): 9.03 (m, 2H), 8.86 (dd, J = 12.6, 8.1 Hz, 2H), 8.81 (dd, J = 6.2, 2.8 Hz, 1H), 8.73 (d, J = 7.2 Hz, 1H), 8.34 (s, 2H), 8.02 – 7.89 (m, 4H), 6.39 (d, J = 24.0 Hz, 2H), 4.34 – 4.26 (m, 1H), 4.16 – 4.09 (m, 1H), 3.42 (s, 2H), 3.11 – 3.04 (m, 1H), 3.00 – 2.90 (m, 1H), 2.90 – 2.76 (m, 3H), 2.57 (d, J = 12.5 Hz, 2H), 2.19 – 2.07 (m, 2H), 2.02 (d, J = 11.5 Hz, 2H), 1.81 – 1.65 (m, 2H), 1.64 – 1.51 (m, 3H), 1.51 – 1.39 (m, 1H), 1.41 – 1.25 (m, 2H).
^{13}C NMR (126 MHz, DMSO-d_6): 178.9, 177.8, 138.5, 138.3, 138.2, 131.2, 131.1, 123.0, 122.5, 61.4, 59.7, 58.6, 55.7, 50.9, 45.8, 45.6, 44.2, 36.6, 28.3, 27.0, 26.1, 26.1, 8.9.
HRMS (ESI positive mode, m/z): calculated for $C_{35}H_{37}N_7NiO_3S$ $[M+H]^+$ 694.2105; found 694.2091.

3. HABA displacement titration for determining the binding constant of [(Biot^{C4}-bAQ)Ni] 1 for Sav WT

HABA titration was performed according to a previously reported procedure.[5-7] In a quartz cuvette, a solution of streptavidin WT (Sav WT, tetrameric, initial concentration 8 µM, 2.4 mL, 0.0192 µmol, 1.0 equiv) in PBS buffer (20 mM, pH 7) was added. A solution of 2-(4'-hydroxyazobenzene)benzoic acid (HABA, 9.6 mM, 300 µL, 2.88 µmol, 150 equiv) in PBS buffer (20 mM, pH 7) was added and the mixture was incubated for 5 min to ensure full saturation of the biotin-binding sites. A blank (PBS buffer only) was measured at 506 nm and the absorbance of the HABA·Sav solution was determined. Aliquots of [(Biot^{C4}-bAQ)Ni] **1** (0.96 mM in DMSO) or biotin (0.96 mM in DMSO) were added to the HABA·Sav solution in 0.50 equiv. step (10 µL per step, up to 5.0 equiv). The CD spectrum (at 506 nm) was recorded 2 minutes after each addition and the molar ellipticity was plotted against the equivalents of [(Biot^{C4}-bAQ)Ni] **1** or biotin added. The decrease of the CD signal ceased once all the HABA was displaced by [(Biot^{C4}-bAQ)Ni] **1** or biotin. The measured data were fitted according to a published procedure.[8, 9]

$$A = (K_a + K_b + C_a + X - C_p)$$

$$B = (K_b * (C_a - C_p) - K_a * (X - C_p) - K_a * K_b)$$

$$\theta = (\arccos\left(\frac{-2*(K_a+K_b+C_a+X-C_p)^3+9*(K_a+K_b+C_a+X-C_p)*(K_b*(C_a-C_p)+K_a*(X-C_p)+K_a*K_b)-27*(-K_a*K_b*C_p)}{2*\mathrm{sqrt}\left(\left((K_a+K_b+C_a+X-C_p)^2-3*(K_b*(C_a-C_p)+K_a*(X-C_p)+K_a*K_b)\right)^3\right)}\right))$$

$$C_{bound} = C_a * (2 * \mathrm{sqrt}(A^\wedge 2 - 3 * B) * \cos(\theta/3) - A)/(3 * K_a + \left(2 * \mathrm{sqrt}(A^2 - 3 * B)\right) * \cos\left(\frac{\theta}{3}\right) - A))$$

$C_{free} = C_a - C_{bound}$

$Y = C_{bound} * E_{bound} + Baseline$

Abbreviations:

$K_a = K_{HABA}$

$K_b = K_{cofactor}$

$C_a = [HABA]_{total}$

$X = [Cofactor]_{total}$

$C_p = [Sav_{mono}]_{total}$

$C_{bound} = [HABA{\cdot}Sav]$

E_{bound} = molar ellipticity

Y = Calculated signal

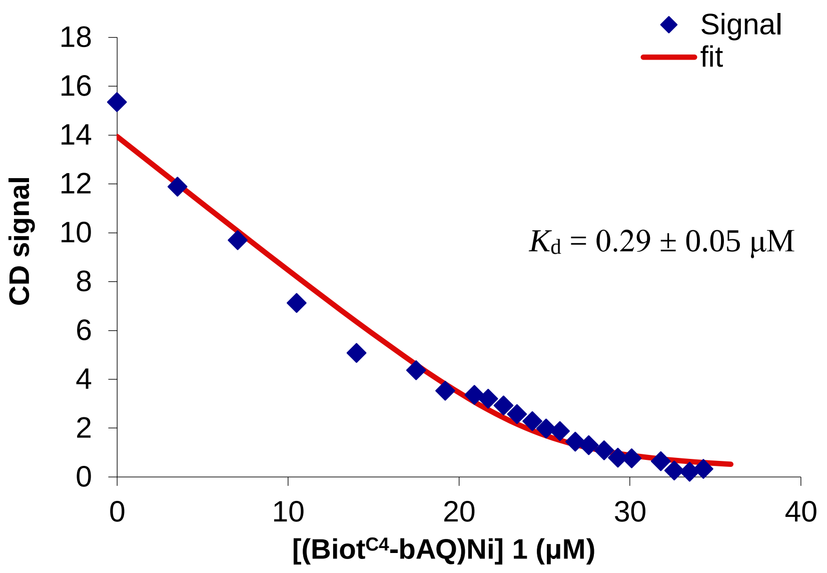

Figure S1. HABA titration experiments with [(Biot^{C4}-bAQ)Ni] **1** (blue diamonds) and fitted curved (red line).

4. Crystallographic characterization of [(Biot^{C4}-bAQ)Ni] 1 · Sav WT

Lyophilized Sav wild-type was dissolved in ultrapure water (18.2 MΩ·cm, MilliQ, Millipore Corporation, Burlington, USA). Sav WT (200 µL of a 10 mg/mL stock solution) was mixed with [(Biot^{C4}-bAQ)Ni] **1** (5 µL of a 10 mg/mL stock solution in DMSO) and Tris/HCl buffer (4 µL of a 1M solution) immediately before crystallization. For sitting drop vapor diffusion, the protein solution (0.15 µL) was mixed in a 1:1 ratio with the precipitation buffer (0.2 M sodium acetate, 0.1 M Tris, pH 8.5, 30% w/v PEG 4K). The drop was equilibrated against a reservoir of the precipitation buffer (32 µL at 20 °C). Crystals of [(Biot^{C4}-bAQ)Ni] **1** ·Sav WT grew within 4 days. The crystals were cryo-protected with 25% ethylene glycol and flash-frozen in liquid nitrogen prior to data collection.

The data collection was carried out at the Swiss Light Source beam line PSI at a wavelength of 1.0000 Å. XDS[10] was used for crystal indexing, integration and AIMLESS[11] for scaling, within the graphical interface CCP4i2[12] of the CCP4 suite. The structures were solved by molecular replacement using PHASER-MR[13] and the streptavidin structure PDB:7ZOF as search model. Refinement was carried out by REFMAC5[14] and for structure modeling and electron-density visualization COOT[15] was used. Ligand restraints were generated using eLBOW[16]. Figures were generated with PyMOL (the PyMOL Molecular Graphics System, Version 2.5.0, Schrödinger, LLC). Data collection and refinement statistics are listed in **Table S1**. The data have been deposited under PDB ID 8QQ3.

Four monomers are present in the asymmetric unit (corresponding to the homotetrameric Sav WT structure, space group C121). Residual electron density in the F_o-F_c map was observed in the biotin-binding site of streptavidin for all four subunits, in molecule A-C the electron density around the cofactor was strong whereas in molecule D the electron density was weaker. Furthermore, anomalous dispersion density was observed. Modeling of the cofactor [(Biot^{C4}-bAQ)Ni] **1** into the electron density projected the nickel in the position of the anomalous density peak. Finally, in three of the four Sav subunits the cofactor was modelled.

Table S1. Data processing and crystal structure refinement statistics

Sav	Sav WT
Cofactor	[(Biot^{C4}-bAQ)Ni] **1**
PDB Code	8QQ3

Data Processing Statistics

Resolution Range (Å)	46.13-1.60 (1.66-1.60)
Cell Parameters<br>- a, b, c (Å)<br>- α, β, γ (°)	115.03, 88.71, 57.79<br>90.00, 97.13, 90.00
Space group	C121
Unique reflections	75827 (7553)
Rmerge (%)	6.7 (123.6)
Multiplicity	6.9 (7.0)
Mean I/Sig(I)	14.5 (1.1)
Completeness (%)	99.9 (99.7)
CC (1/2)	0.999 (0.735)

Structure Refinement Statistics

R_{work}/R_{free}	0.17/0.20
RMS deviation<br>-Bond length (Å)<br>-Bond angles (°)<br>-Ramachandran favored (%)	 0.008<br>1.00<br>98.0
Average B-factors (Å^2)<br>-Protein<br>-Ligands<br>-Solvent	 36.4<br>48.2<br>46.9

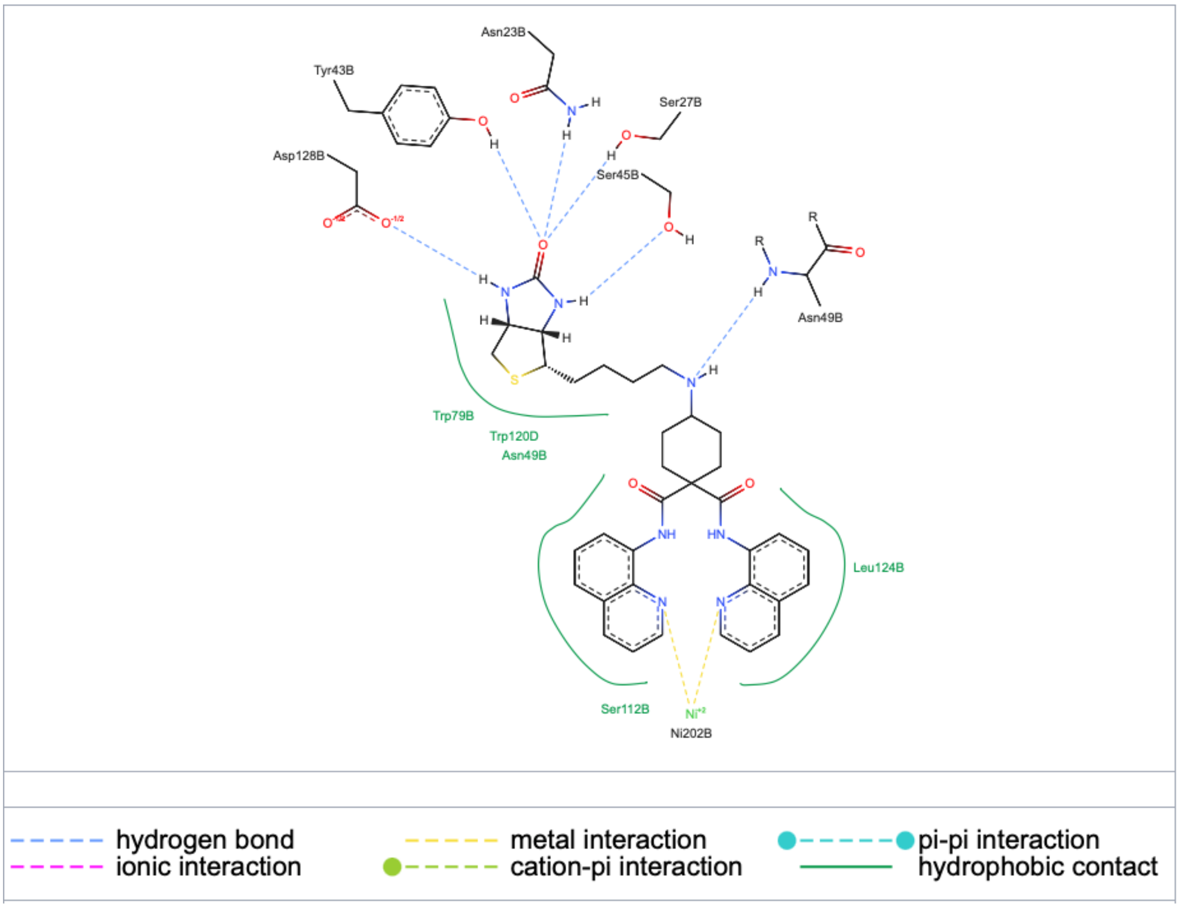

Figure S2. Schematic representation of the interactions between Sav WT and cofactor [(Biot^{C4}-bAQ)Ni] **1.**

5. Expression and purification of Sav mutants

5.1. General procedure for the expression and purification of Sav variants tested in catalysis

The plasmids of the Sav mutants were produced according to our previous procedure.[4] Plasmids were transformed into *E. coli* BL21 (DE3) chemically-competent cells. After heat-shock at 42 °C, the cells were placed on ice for 2 min. Super Optimal Broth medium (SOC, 500 µL) was added into vials, and the cells were incubated for 1 h on a shaker (300 rpm at 37 °C). The suspension (150 µL) was plated out on Lysogeny Broth (LB) agar plates, supplemented with antibiotic (50 µg/mL kanamycin). The plates were incubated (37 °C, 16 h). The preculture was prepared by inoculating fresh single colony in 2.5 mL LB medium in 24-well plates and incubated (37 °C, 300 rpm, 16 h). The preculture (2.0 mL) was inoculated in the main culture (200 mL, ZYP-5052 medium containing 200 µg/mL kanamycin) and further incubated (25 °C, 200 rpm, 24 h). The cells were harvested by centrifugation (4 °C, 3500 g, 10 min) and frozen overnight at -20 °C. The cells were lysed for 2 h. The suspension was frozen again for 5 h and mixed with twice-concentrated IBB buffer (40 mL). The samples were centrifuged (4 °C, 4200

g, 25 min), and the clear supernatant was collected and subsequently loaded on iminobiotin sepharose beads column for affinity purification. The column was washed with IBB buffer (10 column volumes) and then eluted with 0.1% HOAc solution in Milli-Q water three times to afford purified Sav variants. Then, the solvent was exchanged to phosphate buffer (pH 9, 100 mM). Finally, the concentration of purified Sav variants was determined by nanodrop.

ZYP-5052 medium composition: salts: KH_2PO_4 (50 mM), Na_2HPO_4 (50 mM), $(NH_4)_2SO_4$ (5 mM); sugars: glucose monohydrate (2.77 mM), lactose (6.13 mM) with glycerol (0.5%); yeast tryptone mix (5 g/L yeast extract, 10 g/L tryptone); $MgSO_4$ (1 mM).

Lysis buffer composition: Lysozyme (1 mg/mL) and DNAse I in Tris(HCl) buffer (20 mM, pH 7.5).

IBB buffer composition: $NaHCO_3$ (50 mM, pH 10.8) and NaCl (500 mM).

5.2. Sequence of selected Sav muntants

Amino acid sequence of Sav K112Y:
(M)ASMTGGQQMGRDQAGITGTWYNQLGSTFIVTAGADGALTGTYESAVGNAESRY
VLTGRYDSAPATDGSGTALGWTVAWKNNYRNAHSATTWSGQYVGGAEARINTQW
LLTSGTTEANAWYSTLVGHDTFTKVKPSAASIDAAKKAGVNNGNPLDAVQQ

Amino acid sequence of Sav S112F-K121Y:
(M)ASMTGGQQMGRDQAGITGTWYNQLGSTFIVTAGADGALTGTYESAVGNAESRY
VLTGRYDSAPATDGSGTALGWTVAWKNNYRNAHSATTWSGQYVGGAEARINTQW
LLTFGTTEANAWYSTLVGHDTFTKVKPSAASIDAAKKAGVNNGNPLDAVQQ

Amino acid sequence of Sav S112P-K121Y:
(M)ASMTGGQQMGRDQAGITGTWYNQLGSTFIVTAGADGALTGTYESAVGNAESRY
VLTGRYDSAPATDGSGTALGWTVAWKNNYRNAHSATTWSGQYVGGAEARINTQW
LLTPGTTEANAWYSTLVGHDTFTKVKPSAASIDAAKKAGVNNGNPLDAVQQ

6. Catalysis with purified Sav variants

6.1. General procedure for catalysis with purified Sav variants

To a 1.5 mL HPLC glass vial, a stock solution of purified Sav (tetrameric, initial concentration 43.1 μM, 290 μL) in phosphate buffer (0.1 M, pH 9) was added. Then, a stock solution of Ni-cofactor (initial concentration 1.25 mM, 20 μL in MeCN/H_2O = 1:1) was added and the mixture was incubated at room temperature for 5 min. The substrate (initial concentration 250 mM, 40 μL in MeCN) and more MeCN (150 μL) were added successively. The vial was sealed and incubated with a thermoshaker for 1 h (25 ^oC, at 750 rpm). A solution of internal standard (naphthalene, initial concentration 2 mM, 200 μL) in EtOAc (EA) was added and the mixture was extracted with EA (HPLC grade, 2 × 300 μL). The combined organic layers were dried over $MgSO_4$. The extract was filtered and analyzed by GC-MS with an Agilent HP-5 column (30 m × 0.25 mm × 0.25 μm). Retention time: chlorocyclohexane: 2.6 min; internal standard (naphthalene): 13.7 min.

GC methods:

Column: Agilent HP-5 column (30 m × 0.25 mm × 0.25 μm);

Inlet mode: Split with a split ratio: 20:1;

Carrier gas: helium (Total Flow: 48.1 mL/min; Column Flow: 2.0 mL/min)

Column oven temperature: 70 ^oC (isothermal mode for 15 min)

Table S2. Results of ANCase screening with Sav mutants

Sav variants	yield (%)	TON
w/o Sav	5.2	21
WT	12.7	51
S112A	14.3	57
S112V	10.7	43
S112I	15.2	61
S112L	11.3	45
S112M	9.4	37
S112F	10.6	42
S112Y	10.4	41
S112W	9.8	39
S112N	13.3	53
S112Q	8.2	33

S112T	12.9	51
S112D	14.2	57
S112E	6.2	25
S112H	11.6	46
S112K	10.7	43
S112R	15.2	61
S112C	14.5	58
S112G	11.5	46
S112P	15.1	61
K121A	12.4	50
K121V	13.3	53
K121I	13.7	55
K121L	12.7	51
K121M	11.7	47
K121F	17.0	68
K121Y	18.1	72
K121W	15.2	61
K121N	15.7	63
K121Q	13.4	53
K121S	12.3	49
K121T	13.5	54
K121D	15.7	63
K121E	10.9	44
K121H	14.2	57
K121R	10.8	43
K121C	11.7	47
K121G	13.5	54
K121P	17.7	71
S112A-K121Y	20.9	84
S112V-K121Y	20.6	82
S112I-K121Y	16.0	64
S112L-K121Y	16.2	65
S112M-K121Y	16.5	66

S112F-K121Y	22.9	92
S112Y-K121Y	14.9	59
S112N-K121Y	14.7	59
S112Q-K121Y	13.8	55
S112S-K121Y	18.1	72
S112T-K121Y	16.4	66
S112E-K121Y	19.5	78
S112H-K121Y	15.7	63
S112K-K121Y	13.2	53
S112R-K121Y	13.9	55
S112C-K121Y	18.8	75
S112P-K121Y	21.5	86

7. Supporting tables

Table S3. Screening of purified Sav variants for the chlorination of cyclopentane[a]

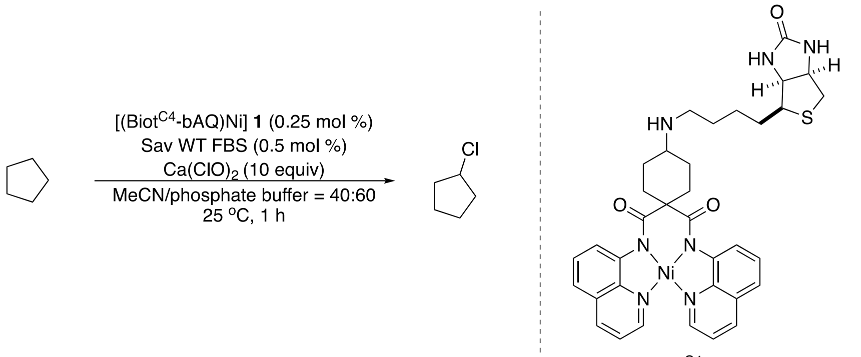

[(Biot^{C4}-bAQ)Ni] **1**

entry	Sav variants	TON
1	WT	42
2	K121Y	33
3	S112F-K121Y	26
4	S112P-K121Y	56±2

[a]Reaction conditions: [substrate] = 20 mM, [$Ca(ClO)_2$] = 200 mM, [[(Biot^{C4}-bAQ)Ni] **1**] = 50 μM, [Sav FBS] = 100 μM, MeCN/phosphate buffer (pH 9, 100 mM) = 200/300 μL, 25 °C, 1 h.

Table S4. Screening of purified Sav variants for the chlorination of toluene[a]

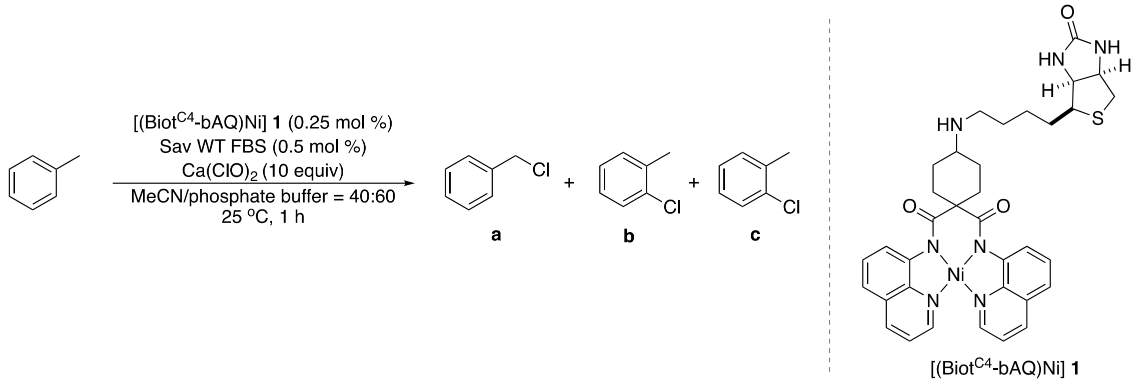

entry	Sav variants	TON			TTON
		**a**	**b**	**c**	
1	WT	78	3	2	83
2	K121Y	84	4	2	90
3	S112F-K121Y	100±7	16±4	9±2	125
4	S112P-K121Y	85	11	6	102

[a]Reaction conditions: [substrate] = 20 mM, [Ca(ClO)$_2$] = 200 mM, [[(Biot^{C4}-bAQ)Ni] **1**] = 50 μM, [Sav FBS] = 100 μM, MeCN/phosphate buffer (pH 9, 100 mM) = 200/300 μL, 25 °C, 1 h.

Table S5. Screening of purified Sav variants for the chlorination of ethylbenzene[a]

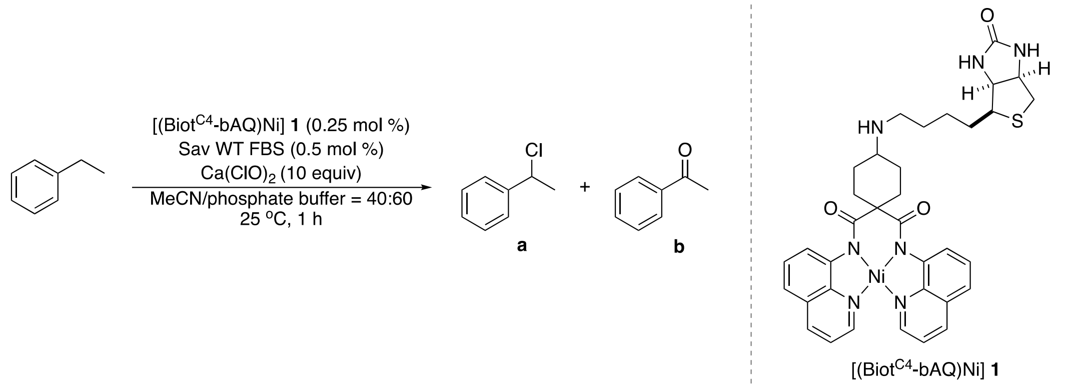

entry	Sav variants	TON		TTON
		a	b	
1	WT	55	190	245
2	K121Y	50	133	183
3	S112F-K121Y	55±4	200±2	255
4	S112P-K121Y	38	191	229

[a]Reaction conditions: [substrate] = 20 mM, [$Ca(ClO)_2$] = 200 mM, [[(Biot^{C4}-bAQ)Ni] **1**] = 50 μM, [Sav FBS] = 100 μM, MeCN/phosphate buffer (pH 9, 100 mM) = 200/300 μL, 25 °C, 1 h.

Table S6. Screening of purified Sav variants for the chlorination of norbornane[a]

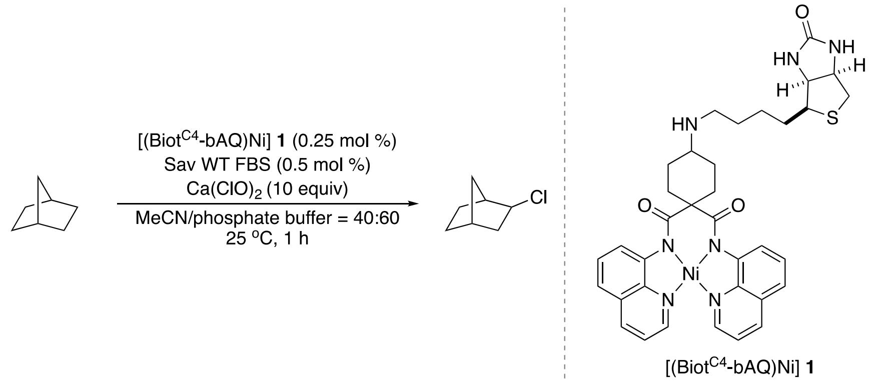

entry	Sav variants	TON
1	WT	44
2	K121Y	46
3	S112F-K121Y	50
4	S112P-K121Y	51±3

[a]Reaction conditions: [substrate] = 20 mM, [$Ca(ClO)_2$] = 200 mM, [[(Biot^{C4}-bAQ)Ni] **1**] = 50 μM, [Sav FBS] = 100 μM, MeCN/phosphate buffer (pH 9, 100 mM) = 200/300 μL, 25 ^{o}C, 1 h.

Table S7. Screening of purified Sav variants for the chlorination of adamantane[a]

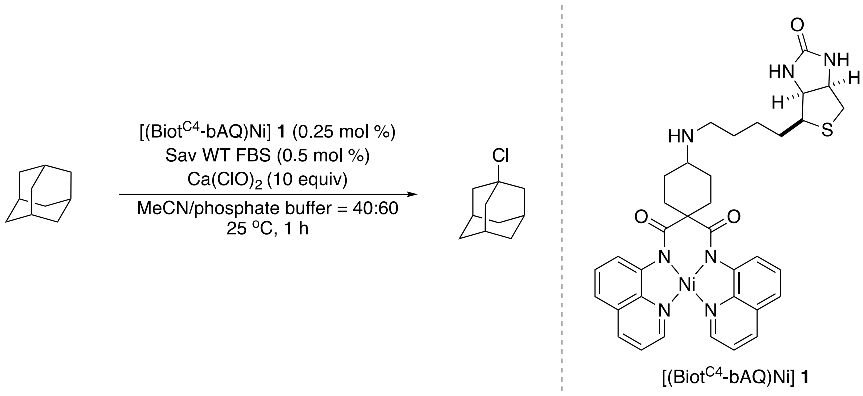

entry	Sav variants	TON
1	WT	11±1
2	K121Y	7
3	S112F-K121Y	8
4	S112P-K121Y	8

[a]Reaction conditions: [substrate] = 20 mM, [$Ca(ClO)_2$] = 200 mM, [[(Biot^{C4}-bAQ)Ni] **1**] = 50 μM, [Sav FBS] = 100 μM, MeCN/phosphate buffer (pH 9, 100 mM) = 200/300 μL, 25 °C, 1 h.

8. Supporting Figures

(a) (b) (c)

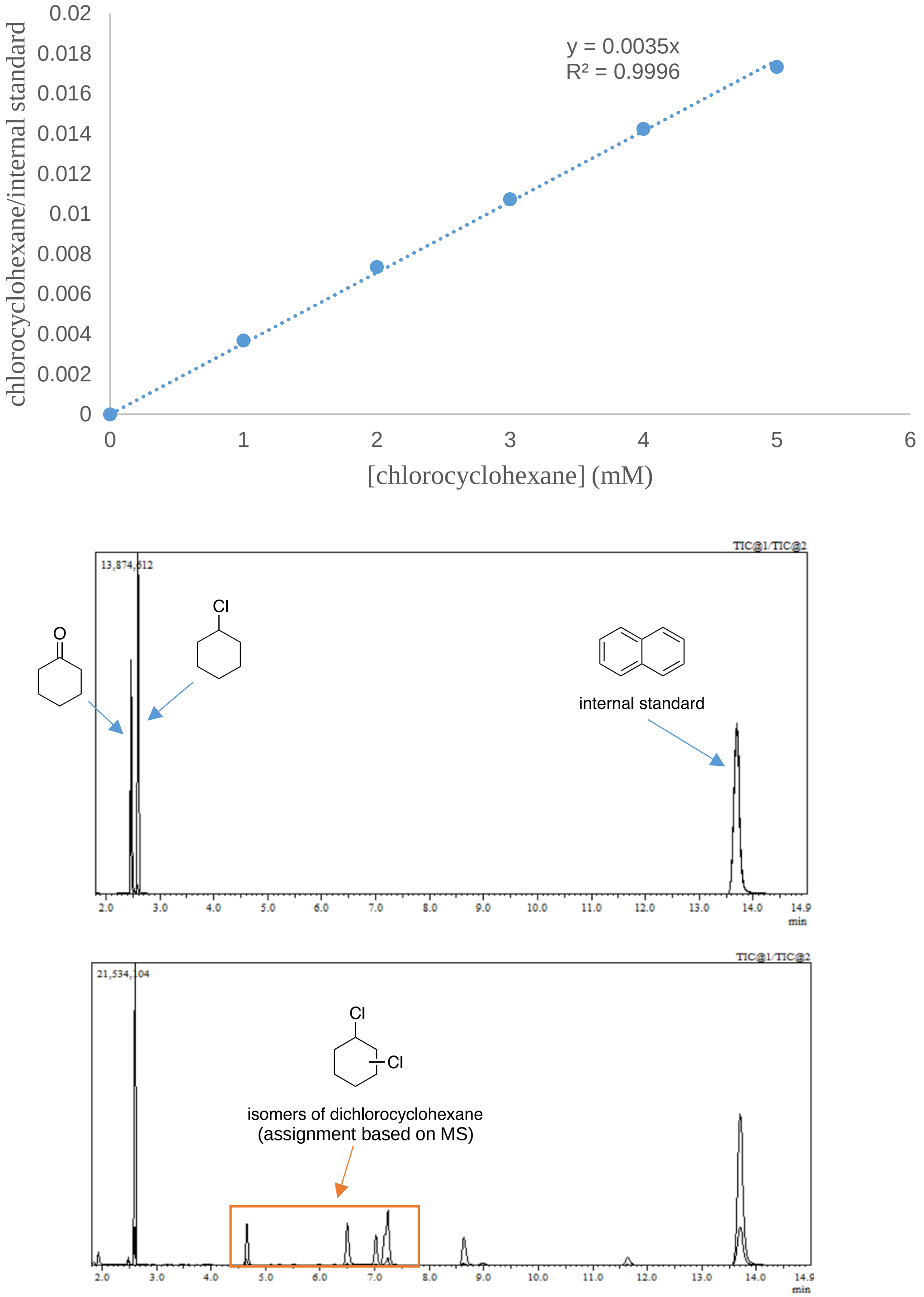

Quantitative Result Table

ID#	Name	R.Time	m/z	Area
1	ketone	2.489	98.00	90298
2	product	2.601	118.00	342016
3	naphthalene	13.703	128.00	8494265

Figure S3. (a) Calibration curve used to determine TON for chlorocyclohexane, using naphthalene as internal standard. (b) GC-MS analysis of a mixture containing commercially available chlorocyclohexane, cyclohexanon and naphthalene, used as a sample for creating the calibration curve. (c) GC-MS analysis resulting from a reaction giving highest TON catalyzed

by [(Biot^{C4}-bAQ)Ni] **1** · Sav S112F-K121Y. GC-MS method: Column: Agilent HP-5 column (30 m × 0.25 mm × 0.25 μm); Inlet mode: Split; Split ratio: 20:1; Carrier gas: helium (Total Flow: 48.1 mL/min; Column Flow: 2.0 mL/min) Column oven temperature: 70 ^{o}C (isothermal mode for 15 min). Internal standard: naphthalene (initial concentration: 2 mM). In the provided GC-MS spectra, two traces are observed. These correspond to the detection of selected ion monitoring (SIM) at m/z 98 (corresponding for cyclohexanone, the upper trace) and 118 (corresponding for chlorocyclohexane, the lower trace) respectively.

(a)

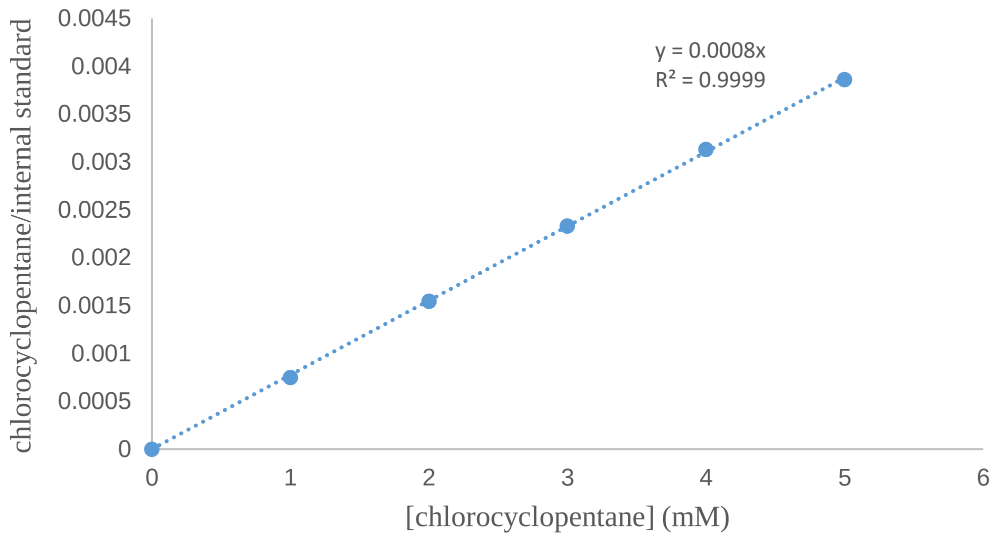

(b)

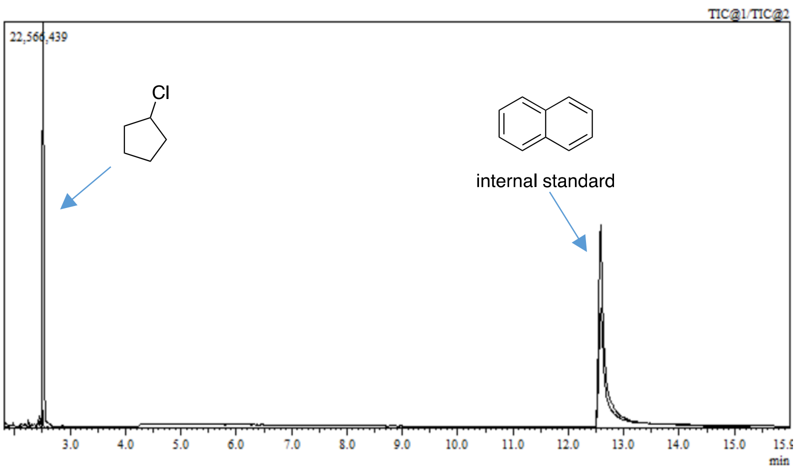

(c)

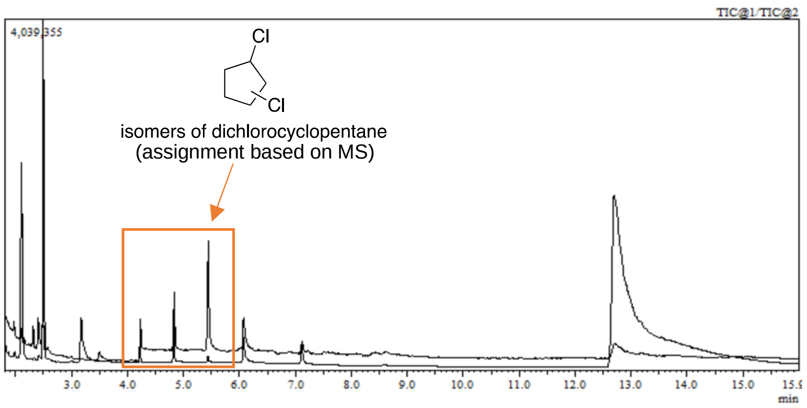

Quantitative Result Table

ID#	Name	R.Time	m/z	Area
1	pro	2.500	104.00	7941
2	naphthalene	12.706	128.00	1359272

Figure S4. (a) Calibration curve used to determine TON for chlorocyclopentane, using naphthalene as internal standard. (b) GC-MS analysis of a mixture containing commercially available chlorocyclopentane and naphthalene, used as a sample for creating the calibration curve. (c) GC-MS analysis resulting from a reaction giving highest TON catalyzed by [(Biot^{C4}-

bAQ)Ni] **1** · Sav S112P-K121Y. GC-MS method: Column: Agilent HP-5 column (30 m × 0.25 mm × 0.25 μm); Inlet mode: Split; Split ratio: 20:1; Carrier gas: helium (Total Flow: 49.0 mL/min; Column Flow: 2.05 mL/min) Column oven temperature: 0 – 3 min: 50 ºC; 3 – 6 min: 50 – 80 ºC; 6 – 16 min: 80 ºC. Internal standard: naphthalene (initial concentration: 1 mM). Retention time: chlorocyclopentane: 2.5 min; internal standard (naphthalene): 12.7 min. In the provided GC-MS spectra, two traces are observed. These correspond to the detection of selected ion monitoring (SIM) at m/z 104 (corresponding for chlorocyclopentane, the upper trace) and 128 (corresponding for naphthalene, the lower trace) respectively.

(a)

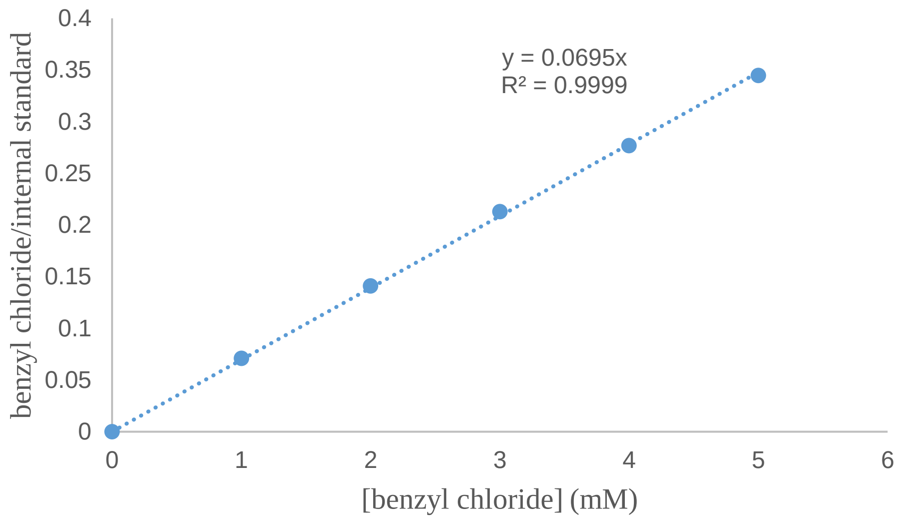
y = 0.0695x
R² = 0.9999
benzyl chloride/internal standard
[benzyl chloride] (mM)
0.4
0.35
0.3
0.25
0.2
0.15
0.1
0.05
0
0
1
2
3
4
5
6

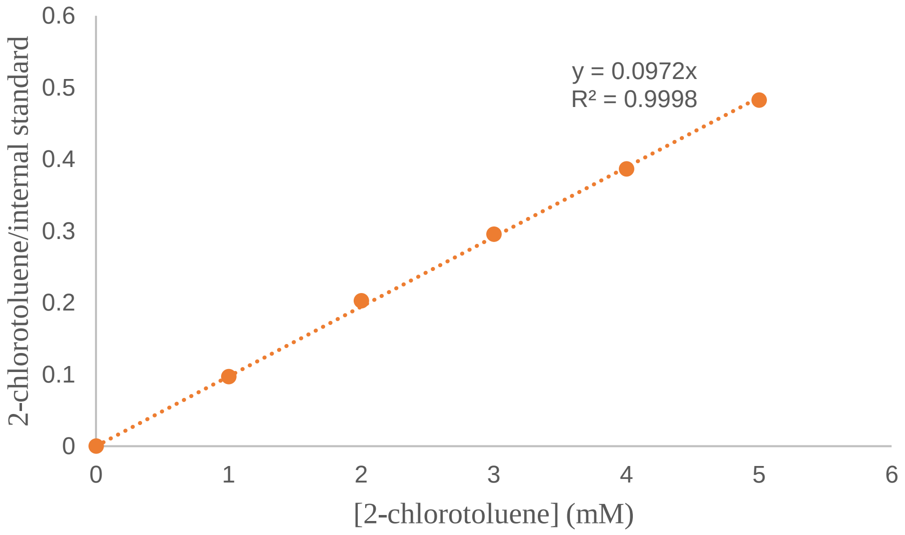
y = 0.0972x
R² = 0.9998
2-chlorotoluene/internal standard
[2-chlorotoluene] (mM)
0.6
0.5
0.4
0.3
0.2
0.1
0
0
1
2
3
4
5
6

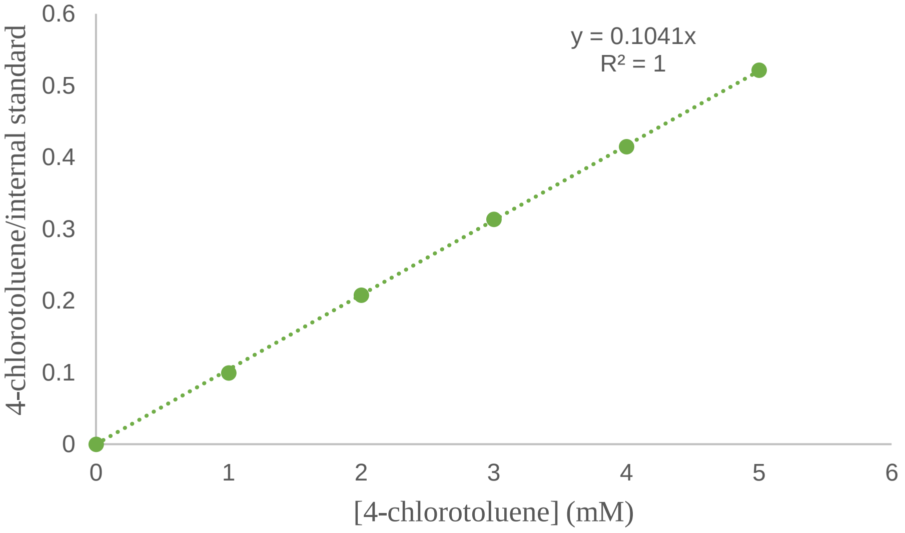
y = 0.1041x
R² = 1
4-chlorotoluene/internal standard
[4-chlorotoluene] (mM)
0.6
0.5
0.4
0.3
0.2
0.1
0
0
1
2
3
4
5
6

(b)

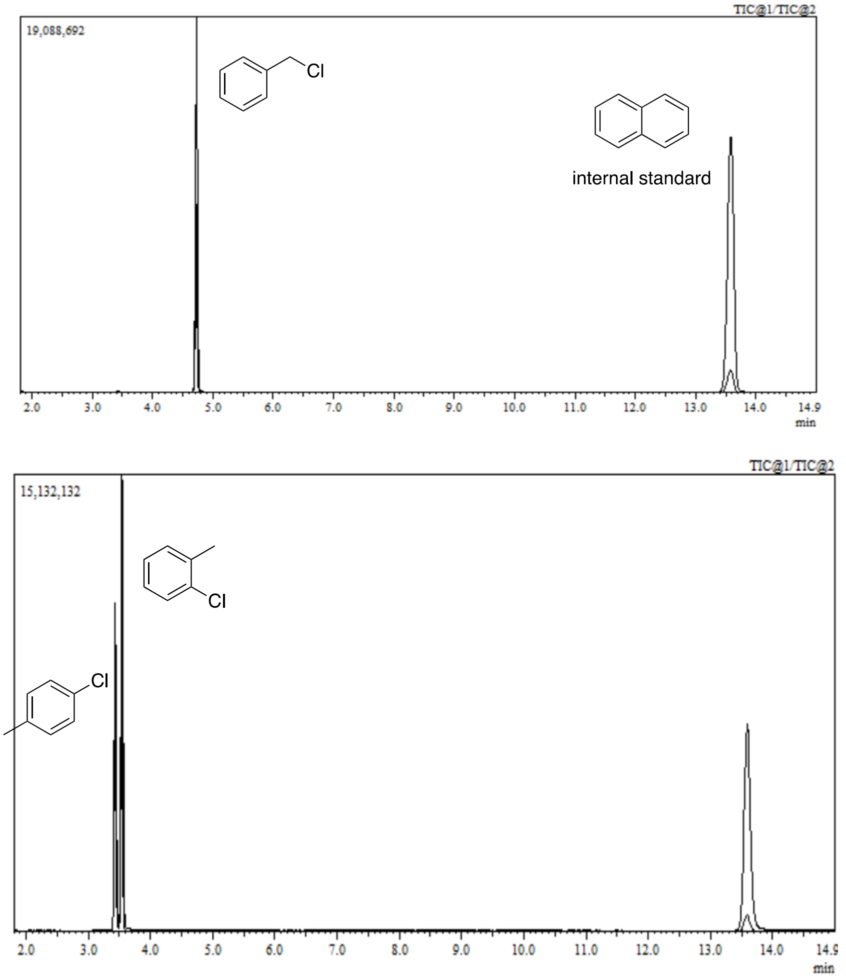

(c)

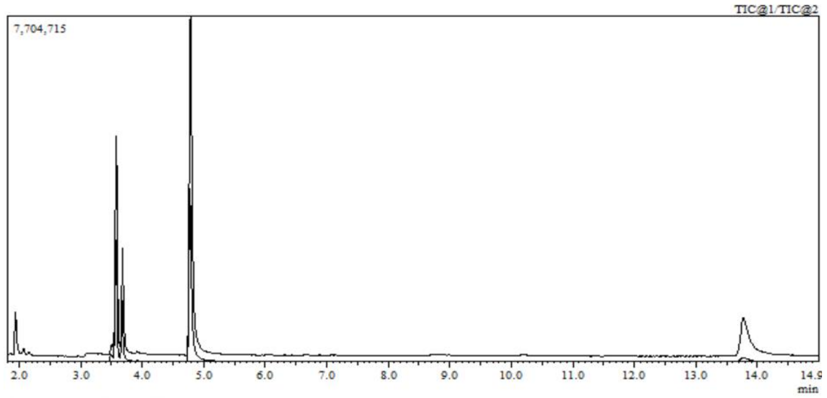

Quantitative Result Table

ID#	Name	R.Time	m/z	Area
1	benzaldehyde	3.505	106.00	280090
2	o-Cl-ph	3.578	126.00	1507161
3	p-Cl-ph	3.679	126.00	881032
4	benzylchloride	5.180	126.00	590
5	naphthalene	13.774	128.00	4713866

Figure S5. (a) Calibration curves used to determine TON for benzyl chloride, 2-chlorotoluene and 4-chlorotoluene, using naphthalene as internal standard. (b) GC-MS analysis of commercially available benzyl chloride, naphthalene; and a mixture containing commercially available 2-chlorotoluene, 4-chlorotoluene and naphthalene, used as samples for creating

corresponding calibration curves. (c) GC-MS analysis resulting from a reaction giving highest TON catalyzed by [(Biot^{C4}-bAQ)Ni] **1** · Sav S112F-K121Y. GC-MS method: Column: Agilent HP-5 column (30 m × 0.25 mm × 0.25 μm); Inlet mode: Split; Split ratio: 20:1; Carrier gas: helium (Total Flow: 48.1 mL/min; Column Flow: 2.0 mL/min) Column oven temperature: 70 ^{o}C (isothermal mode for 15 min). Internal standard: naphthalene (initial concentration: 2 mM). Retention time: benzyl chloride: 5.2 min; 2-chlorotoluene: 3.6 min; 4-chlorotoluene: 3.7 min. internal standard (naphthalene): 13.8 min. In the provided GC-MS spectra, two traces are observed. These correspond to the detection of selected ion monitoring (SIM) at m/z 106 (corresponding for benzyl aldehyde, the upper trace) and 126 (corresponding for the chlorinated products, the lower trace) respectively.

(a)

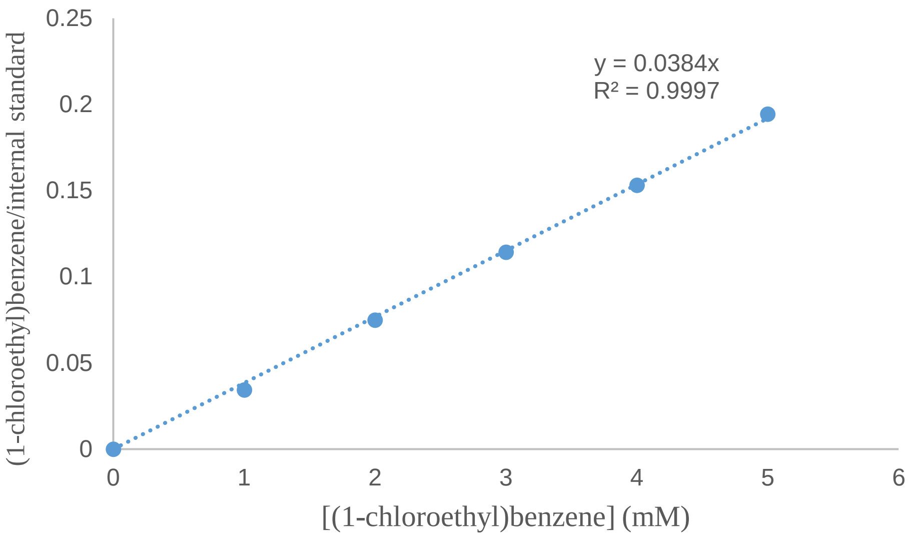
y = 0.0384x
R² = 0.9997
(1-chloroethyl)benzene/internal standard
0.25
0.2
0.15
0.1
0.05
0
0
1
2
3
4
5
6
[(1-chloroethyl)benzene] (mM)

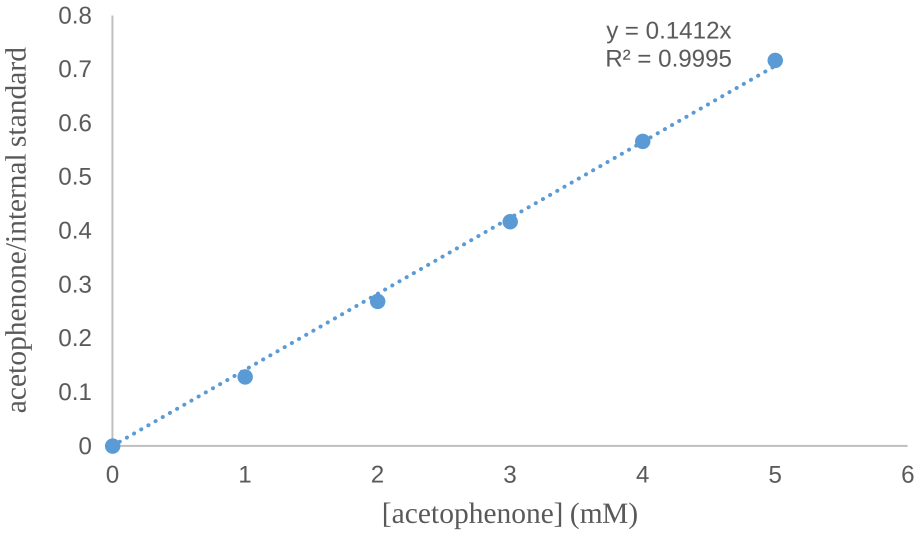
y = 0.1412x
R² = 0.9995
acetophenone/internal standard
0.8
0.7
0.6
0.5
0.4
0.3
0.2
0.1
0
0
1
2
3
4
5
6
[acetophenone] (mM)

(b)

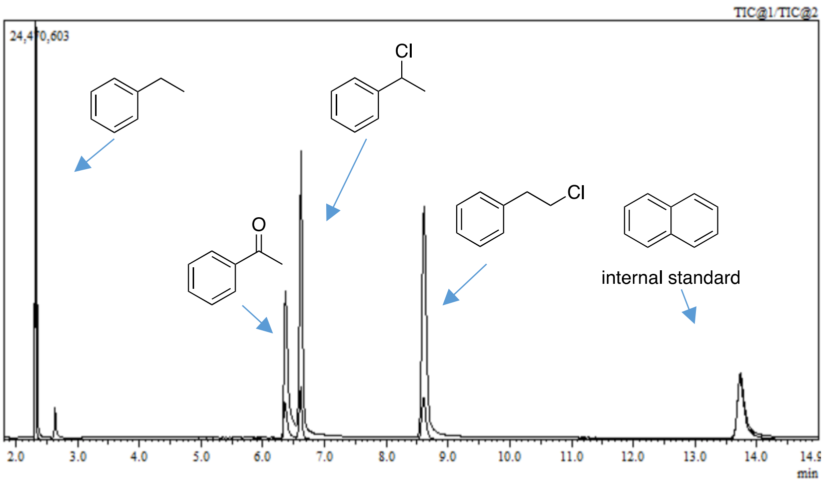
TIC@1/TIC@2
Cl
O
Cl
internal standard
2.0
3.0
4.0
5.0
6.0
7.0
8.0
9.0
10.0
11.0
12.0
13.0
14.0
min

(c)

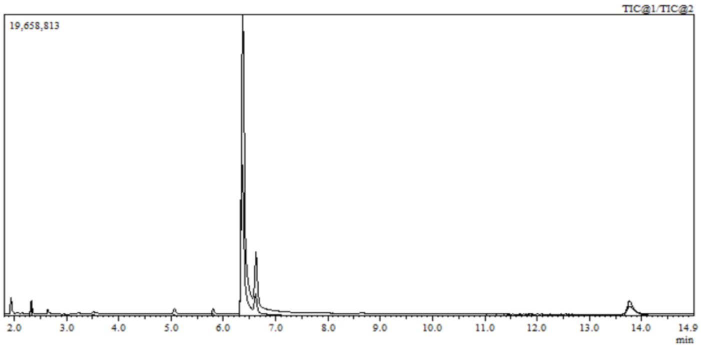

Quantitative Result Table

ID#	Name	R.Time	m/z	Area
1	EB	2.329	106.00	291303
2	ketone	6.367	120.00	10011117
3	chloro-b	6.624	140.00	712287
4	chloro-l	8.646	140.00	38817
5	naphthalene	13.769	128.00	2800618

Figure S6. (a) Calibration curves used to determine TON for (1-chloroethyl)benzene and acetophenone, using naphthalene as internal standard. (b) GC-MS analysis of a mixture containing commercially available ethylbenzene, acetophenone, (1-chloroethyl)benzene, (2-chloroethyl)benzene and naphthalene, used as a sample for creating corresponding calibration curves. (c) GC-MS analysis resulting from a reaction giving the highest TON catalyzed by [(Biot^{C4}-bAQ)Ni] **1** · Sav S112F-K121Y. GC-MS method: Column: Agilent HP-5 column (30 m × 0.25 mm × 0.25 µm); Inlet mode: Split; Split ratio: 20:1; Carrier gas: helium (Total Flow: 48.1 mL/min; Column Flow: 2.0 mL/min) Column oven temperature: 70 ^{o}C (isothermal mode for 15 min). Internal standard: naphthalene (initial concentration: 1 mM). Retention time: (1-chloroethyl)benzene: 6.6 min; acetophenone: 6.4 min. internal standard (naphthalene): 13.8 min. In the provided GC-MS spectra, two traces are observed. These correspond to the detection of selected ion monitoring (SIM) at m/z 106 (corresponding for ethylbenzene, the upper trace) and 120 (corresponding for acetophenone, the lower trace) respectively.

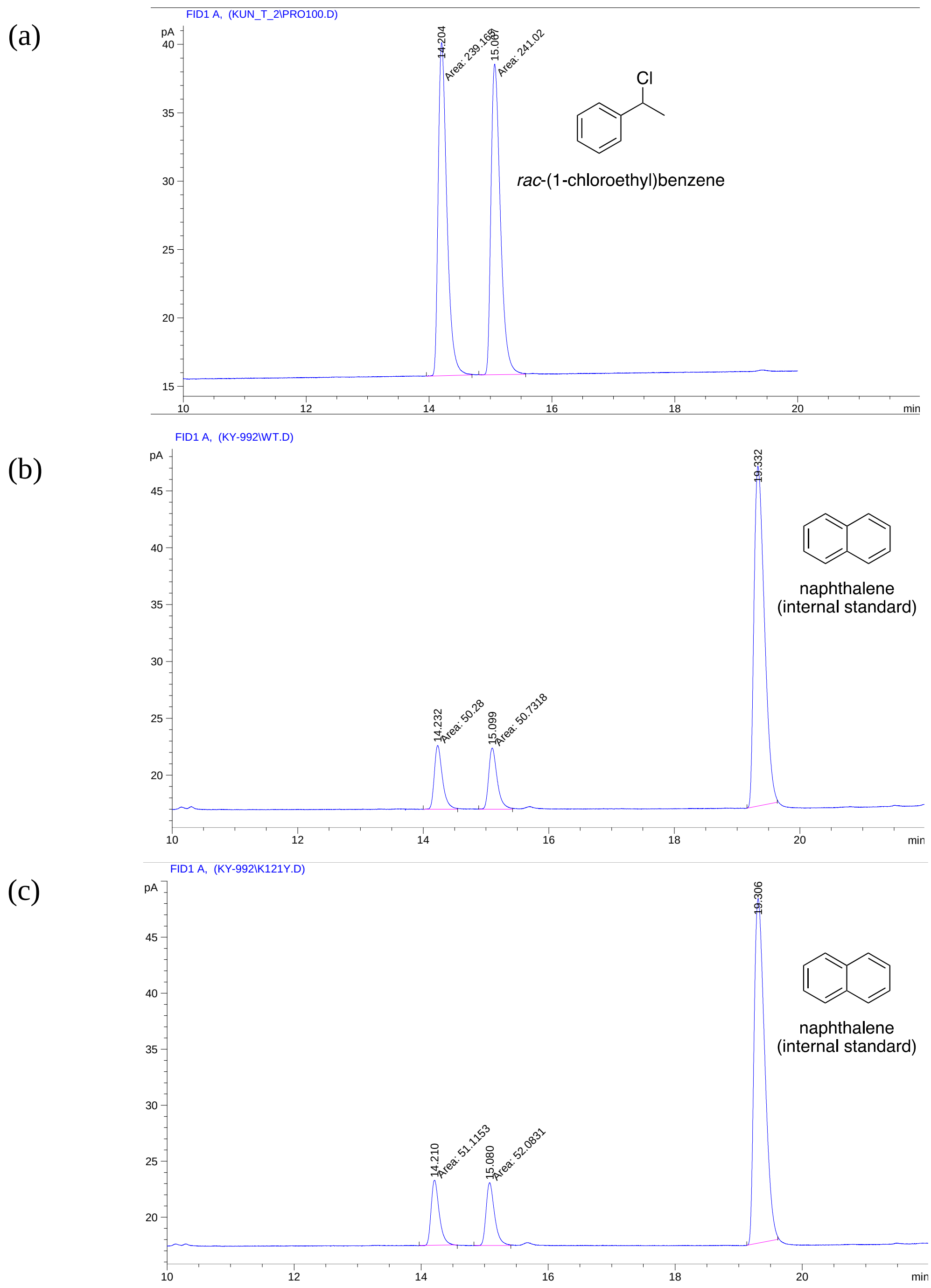

Figure S7. (a) Chiral GC analysis of commercial (*rac*)-(1-chloroethyl)benzene. (b) Chiral GC analysis resulting from a reaction using ethylbenzene as substrate catalyzed by [(Biot^{C4}-bAQ)Ni] **1** · Sav WT. (c) Chiral GC analysis resulting from a reaction using ethylbenzene as substrate catalyzed by [(Biot^{C4}-bAQ)Ni] **1** · Sav K121Y. GC method: column: Astec® CHIRALDEX™ G-TA Capillary GC Column (50 m 0.25 mm 0.12 µm); carrier gas: He; flow: 1.2 mL/min; oven temperature: isothermal, 100 °C for 25 min. Internal standard: naphthalene (initial concentration: 1 mM).

(a)

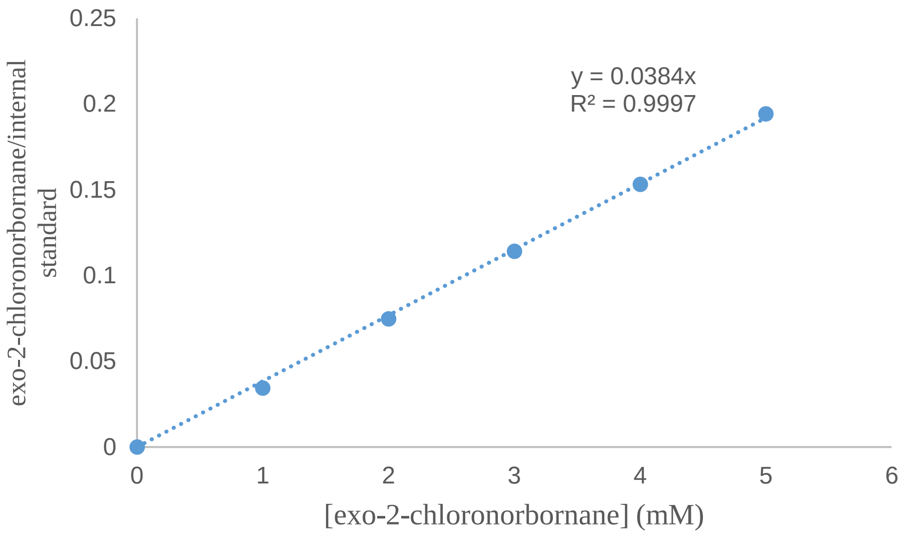

(b)

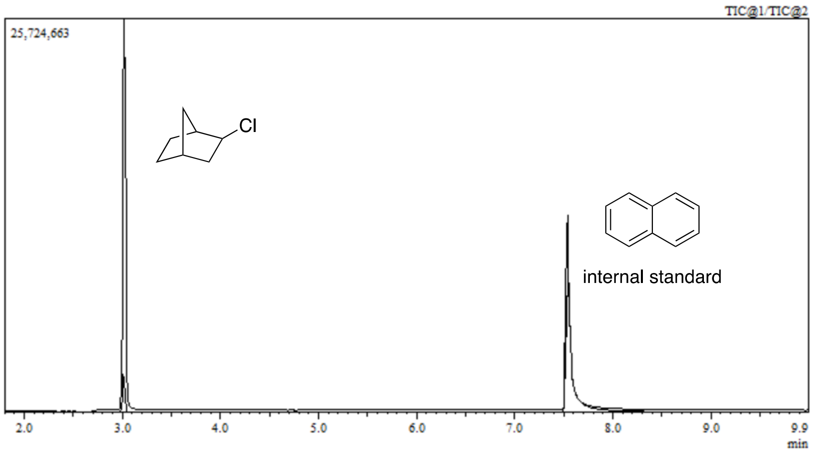

(c)

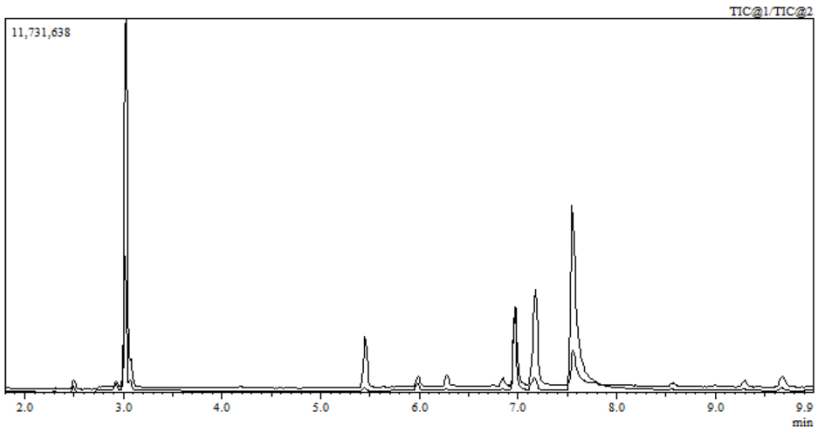

Quantitative Result Table

ID#	Name	R.Time	m/z	Area
1	product	3.025	130.00	659553
2	naphthalene	7.561	128.00	2897937

Figure S8. (a) Calibration curve used to determine TON for exo-2-chloronorbornane, using naphthalene as internal standard. (b) GC-MS analysis of a mixture containing commercially available exo-2-chloronorbornane and naphthalene, used as a sample for creating the calibration curve. (c) GC-MS analysis resulting from a reaction giving the highest TON catalyzed by [(Biot^{C4}-bAQ)Ni] **1** · Sav S112P-K121Y. GC-MS method: Column: Agilent HP-5 column (30

m × 0.25 mm × 0.25 μm); Inlet mode: Split; Split ratio: 20:1; Carrier gas: helium (Total Flow: 47.7 mL/min; Column Flow: 1.98 mL/min) Column oven temperature: 0 – 5 min: 80 ºC; 5 – 7 min: 80 – 100 ºC; 7 – 10 min: 100 ºC. Internal standard: naphthalene (initial concentration: 1 mM). Retention time: exo-2-chloronorbornane: 3.0 min. internal standard (naphthalene): 7.6 min. In the provided GC-MS spectra, two traces are observed. These correspond to the detection of selected ion monitoring (SIM) at m/z 130 (corresponding for exo-2-chloronorbornane, the upper trace) and 128 (corresponding for naphthalene, the lower trace) respectively.

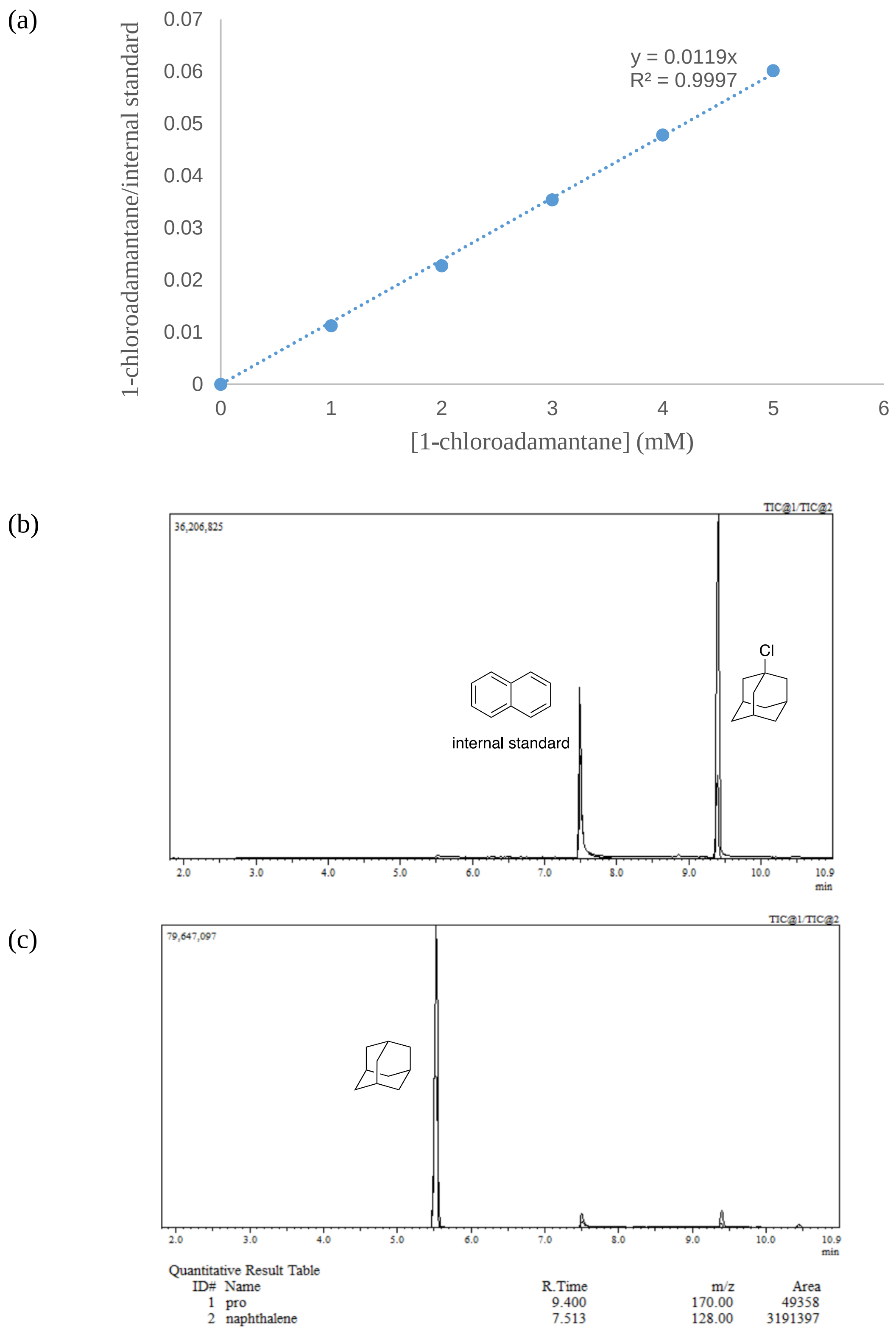

Figure S9. (a) Calibration curve used to determine TON for 1-chloroadamantane, using naphthalene as internal standard. (b) GC-MS analysis of a mixture containing commercially available 1-chloroadamatane and naphthalene, used as a sample for creating the calibration curve. (c) GC-MS analysis resulting from a reaction giving the highest TON catalyzed by [(Biot^{C4}-bAQ)Ni] **1** · Sav WT. GC-MS method: Column: Agilent HP-5 column (30 m × 0.25

mm × 0.25 μm); Inlet mode: Split; Split ratio: 20:1; Carrier gas: Helium (Total Flow: 47.7 mL/min; Column Flow: 1.98 mL/min) Column oven temperature: 0 – 5 min: 80 ºC; 5 – 9 min: 80 – 120 ºC; 9 – 11 min: 120 ºC. Internal standard: naphthalene (initial concentration: 1 mM). Retention time: 1-chloroadamantane: 9.4 min. internal standard (naphthalene): 7.5 min. In the provided GC-MS spectra, two traces are observed. These correspond to the detection of selected ion monitoring (SIM) at m/z 170 (corresponding for 1-chloroadamantane, the upper trace) and 128 (corresponding for naphthalene, the lower trace) respectively.

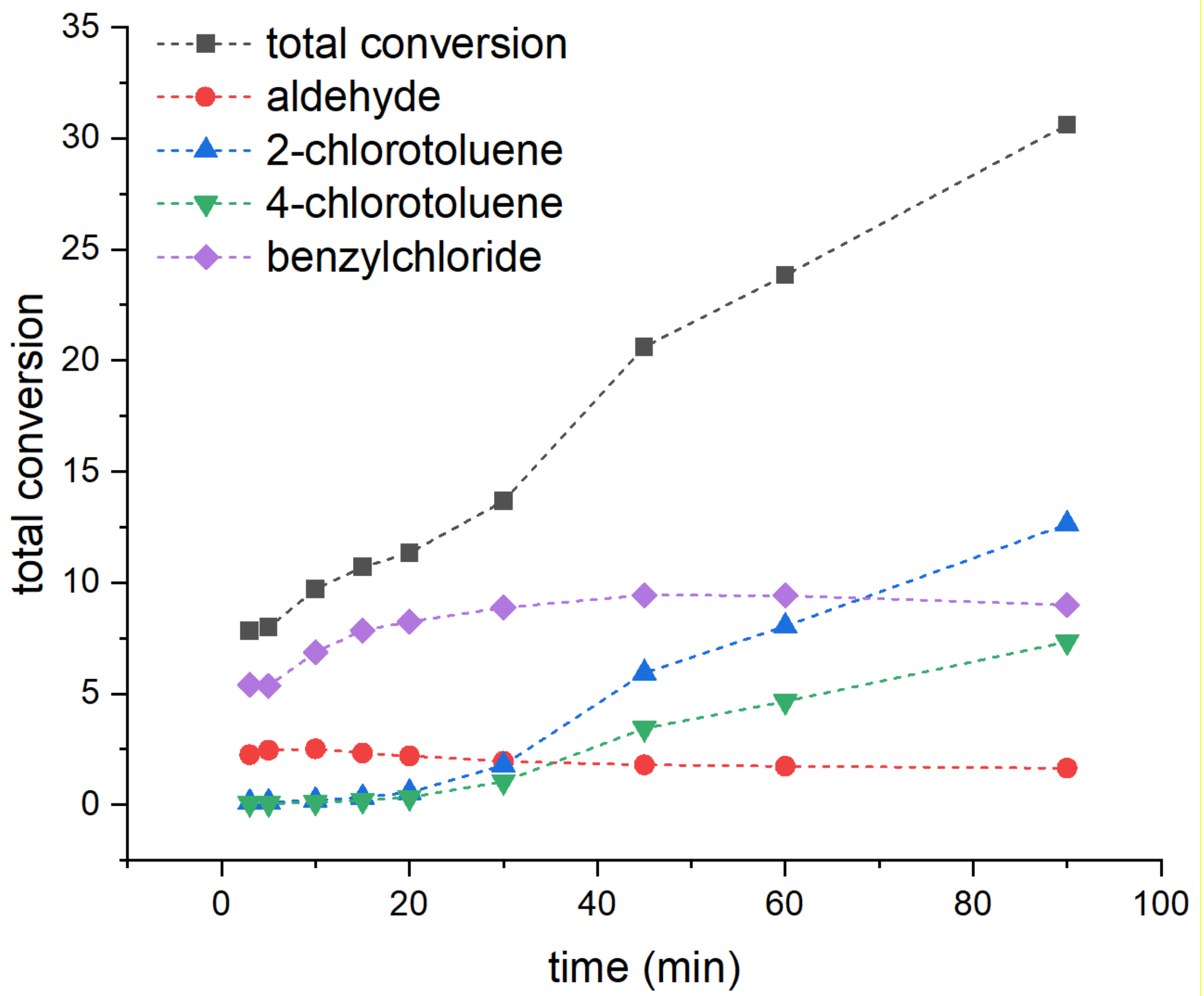

Figure S10. Time-course monitoring of the chlorination of toluene using [(Biot^{C4}-bAQ)Ni] **1** · Sav WT, including the product distribution.

9. NMR Spectra

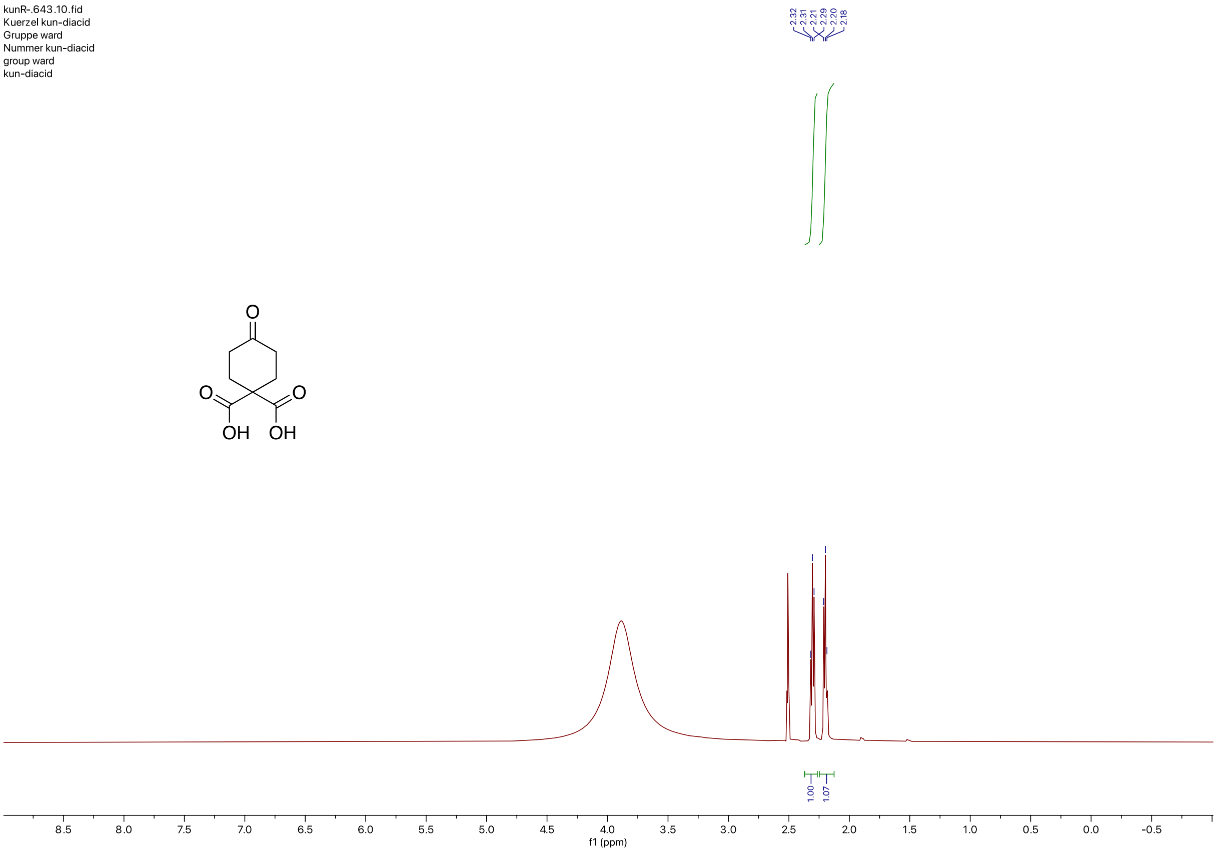

Figure S9. ^{1}H NMR of dicarboxylic acid **S2**.

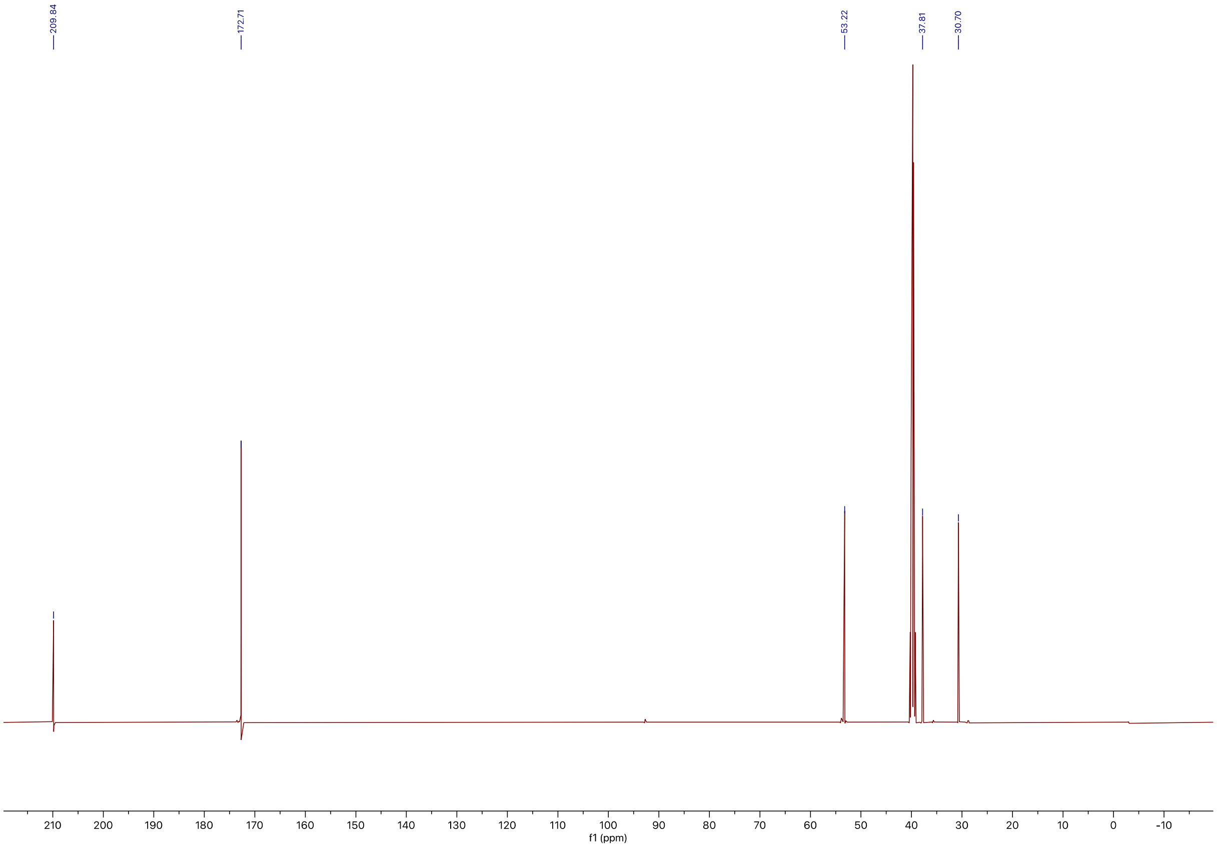

Figure S10. ^{13}C NMR of dicarboxylic acid **S2**.

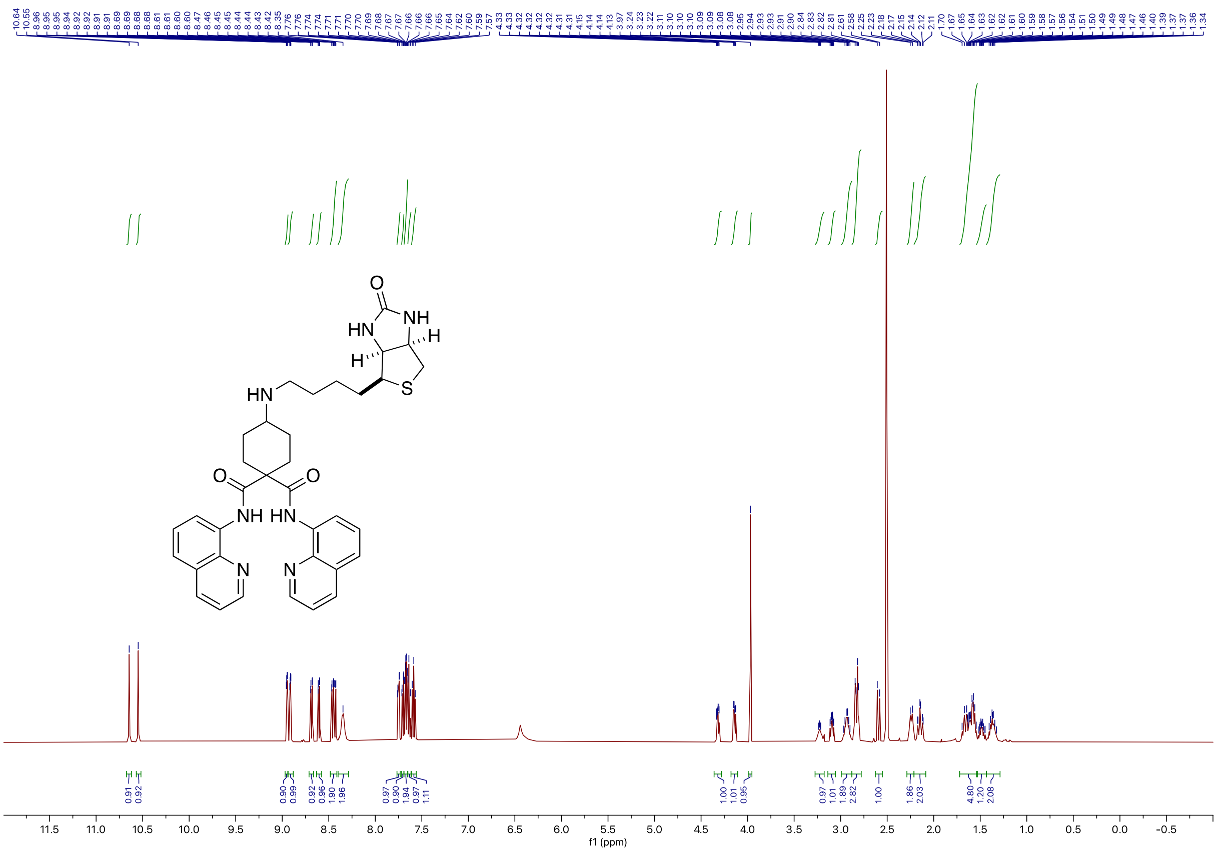

Figure S11. ^{1}H NMR of ligand Biot^{C4}-bAQ **S4**.

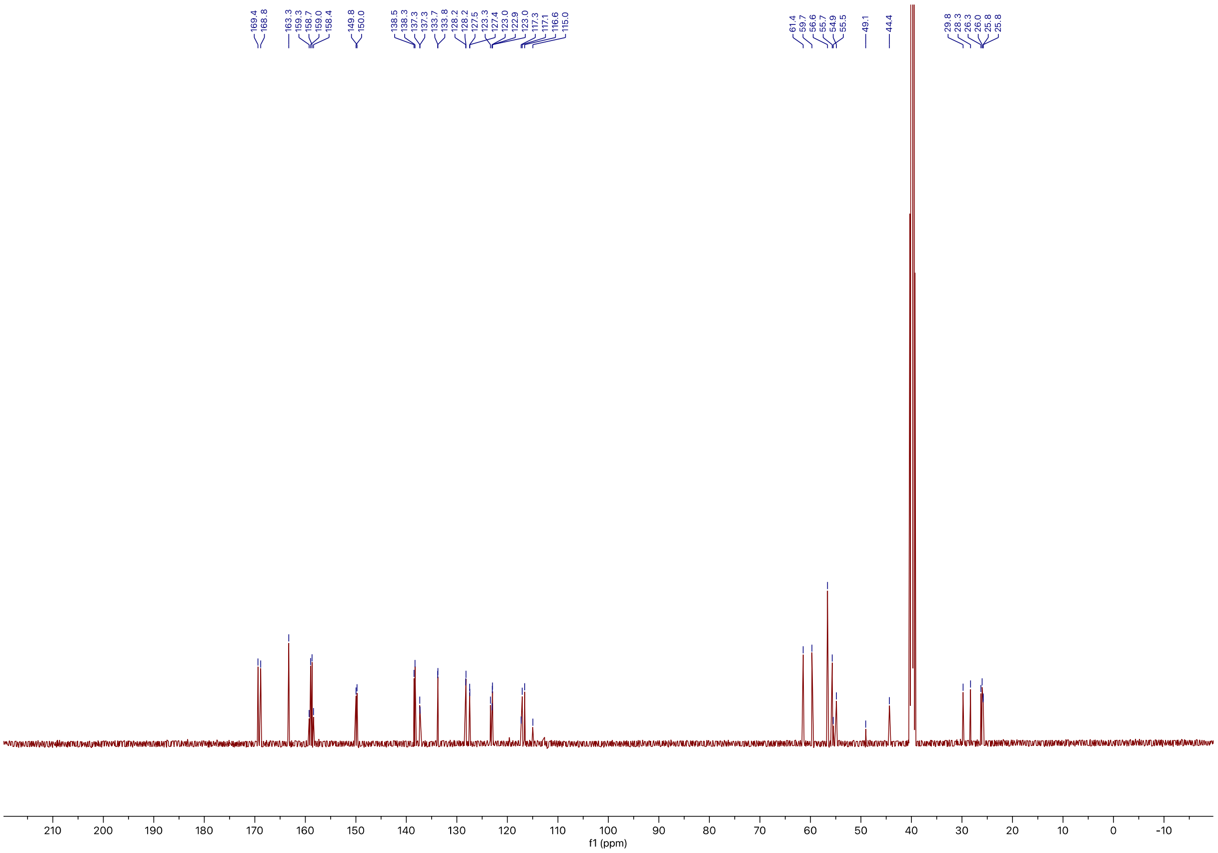

Figure S12. ^{13}C NMR of ligand Biot^{C4}-bAQ **S4**.

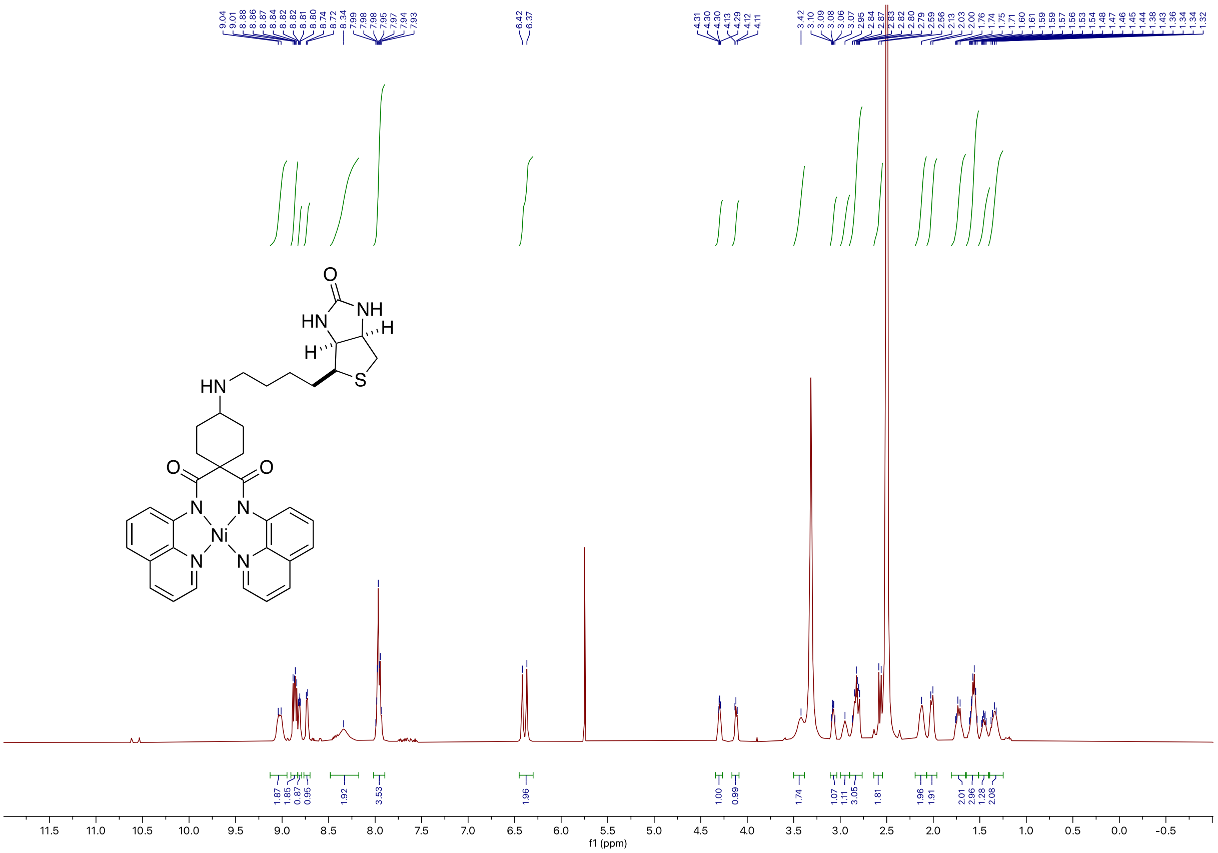

Figure S13. ^{1}H NMR of cofactor [(Biot^{C4}-bAQ)Ni] **1**.

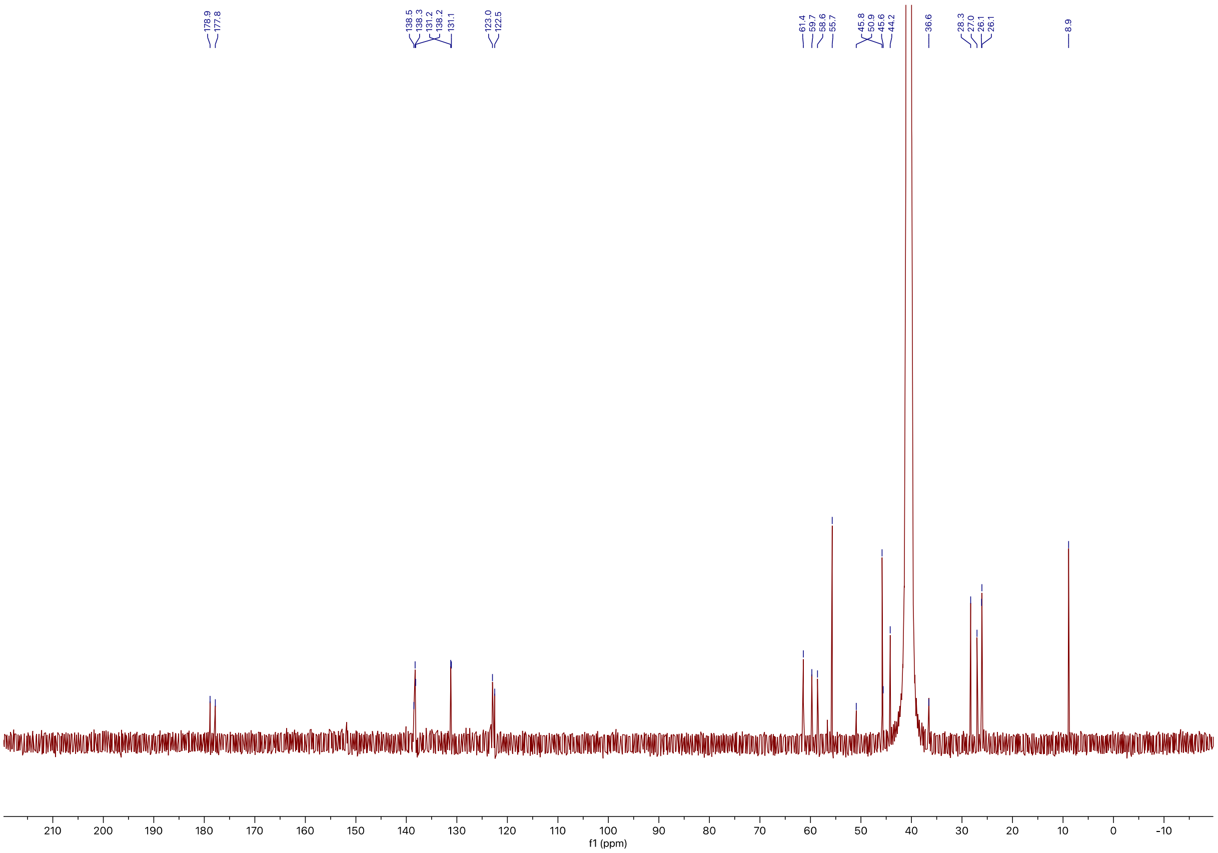

Figure S14. ^{13}C NMR of cofactor [(Biot^{C4}-bAQ)Ni] **1**.

REFERENCES

(1) Lichtenecker, R. J.; Coudevylle, N.; Konrat, R.; Schmid, W. Selective Isotope Labelling of Leucine Residues by Using alpha-Ketoacid Precursor Compounds. *ChemBioChem* **2013**, *14*, 818-821.

(2) Breising, V. M.; Gieshoff, T.; Kehl, A.; Kilian, V.; Schollmeyer, D.; Waldvogel, S. R. Electrochemical Formation of 3,5-Diimido-1,2-dithiolanes by Dehydrogenative Coupling. *Org. Lett.* **2018**, *20*, 6785-6788.

(3) da Costa, T. P. S.; Tieu, W.; Yap, M. Y.; Zvarec, O.; Bell, J. M.; Turnidge, J. D.; Wallace, J. C.; Booker, G. W.; Wilce, M. C. J.; Abell, A. D.; et al. Biotin Analogues with Antibacterial Activity Are Potent Inhibitors of Biotin Protein Ligase. *ACS Med. Chem. Lett.* **2012**, *3*, 509-514.

(4) Adhikari, S.; Sarkar, A.; Dhar, B. B. C-H bond chlorination using nickel(ii) complexes of tetradentate amido-quinoline ligands. *Chem. Commun.* **2022**, *58*, 4075-4078.

(5) Kada, G.; Kaiser, K.; Falk, H.; Gruber, H. J. Rapid estimation of avidin and streptavidin by fluorescence quenching or fluorescence polarization. *Biochim. Biophys. Acta* **1999**, *1427*, 44-48.

(6) Skander, M.; Humbert, N.; Collot, J.; Gradinaru, J.; Klein, G.; Loosli, A.; Sauser, J.; Zocchi, A.; Gilardoni, F.; Ward, T. R. Artificial metalloenzymes: (Strept)avidin as host for enantioselective hydrogenation by achiral biotinylated rhodium-diphosphine complexes. *J. Am. Chem. Soc.* **2004**, *126*, 14411-14418.

(7) Serrano-Plana, J.; Rumo, C.; Rebelein, J. G.; Peterson, R. L.; Barnet, M.; Ward, T. R. Enantioselective Hydroxylation of Benzylic C(sp(3))-H Bonds by an Artificial Iron Hydroxylase Based on the Biotin-Streptavidin Technology. *J. Am. Chem. Soc.* **2020**, *142*, 10617-10623.

(8) Wang, Z.-X. An Exact Mathematical Expression for Describing Competitive Binding of Two Different Ligands to a Protein Molecule. *FEBS Lett.* **1995**, *360*, 111-114.

(9) Rumo, C.; Stein, A.; Klehr, J.; Tachibana, R.; Prescimone, A.; Haussinger, D.; Ward, T. R. An Artificial Metalloenzyme Based on a Copper Heteroscorpionate Enables sp(3) C-H Functionalization via Intramolecular Carbene Insertion. *J. Am. Chem. Soc.* **2022**, *144*, 11676-11684.

(10) Kabsch, W. Xds. *Acta crystallographica. Section D, Biological crystallography* **2010**, *66* (Pt 2), 125-132.

(11) Evans, P. R.; Murshudov, G. N. How good are my data and what is the resolution? *Acta Cryst.* **2013**, *69*, 1204-1214.

(12) Potterton, L.; Agirre, J.; Ballard, C.; Cowtan, K.; Dodson, E.; Evans, P. R.; Jenkins, H. T.; Keegan, R.; Krissinel, E.; Stevenson, K.; et al. CCP4i2: the new graphical user interface to the CCP4 program suite. *Acta Cryst.* **2018**, *74*, 68-84.

(13) Mccoy, A. J.; Grosse-Kunstleve, R. W.; Adams, P. D.; Winn, M. D.; Storoni, L. C.; Read, R. J. Phaser crystallographic software. *J. Appl. Cryst.* **2007**, *40*, 658-674.

(14) Murshudov, G. N.; Skubak, P.; Lebedev, A. A.; Pannu, N. S.; Steiner, R. A.; Nicholls, R. A.; Winn, M. D.; Long, F.; Vagin, A. A. REFMAC5 for the refinement of macromolecular crystal structures. *Acta Cryst.* **2011**, *67*, 355-367.

(15) Emsley, P.; Lohkamp, B.; Scott, W. G.; Cowtan, K. Features and development of Coot. *Acta Cryst.* **2010**, *66*, 486-501.

(16) Moriarty, N. W.; Grosse-Kunstleve, R. W.; Adams, P. D. electronic Ligand Builder and Optimization Workbench (eLBOW): a tool for ligand coordinate and restraint generation. *Acta crystallographica. Section D, Biological crystallography* **2009**, *65* (Pt 10), 1074-1080.
